# Exploring the feasibility and acceptability of an educational intervention on air pollution for healthcare professionals

**DOI:** 10.3389/fpubh.2026.1862924

**Published:** 2026-08-12

**Authors:** Diana Varaden, Ben Hudson, Malcolm White, Camilla Allwood, Lucy G. Anderson

**Affiliations:** 1 Environmental Research Group, School of Public Health, Imperial College London, London, United Kingdom; 2 Kent County Council, Kent, United Kingdom; 3 Global Action Plan, London, United Kingdom; 4 Population Health Sciences, Bristol Medical School, University of Bristol, Bristol, United Kingdom

**Keywords:** air pollution, champion model, clinical education, general practitioners, primary care

## Abstract

**Background:**

Air pollution is the greatest environmental health risk in the UK, contributing to a range of chronic conditions and preventable deaths each year. General Practitioners and wider healthcare professionals (HCPs) are trusted messengers who regularly advise on modifiable risk factors, yet air pollution is largely absent from medical education and patient-facing communications. To address this gap, an online training intervention was developed to equip HCPs with knowledge and confidence to discuss air pollution with patients and act as “champions” cascading their air quality knowledge to colleagues.

**Methods:**

This mixed-methods feasibility study assessed the acceptability of the training intervention, changes in self-reported knowledge and confidence and feasibility of applying a champion model in practise. Outcome measures were underpinned by a Theory of Change. Quantitative data were statistically analysed whilst qualitative data were thematically analysed using a deductive approach.

**Results:**

Forty-three HCPs (40 GPs, a nurse practitioner, a pharmacist and a practise manager) completed the training, with 100% completing the pre-training survey, 90% the post-training survey (*n* = 40) and 53% the three-month follow up (*n* = 26). Improvements were observed across all self-reported outcomes, including understanding of the health impacts of air pollution, the actions patients could take to minimise exposure, and confidence in discussing and advising on the health impacts of air pollution. GPs valued the training and identified a need for practical tools and integration into standard care pathways. Interviews highlighted key barriers to implementing the champion model, including limited time and a lack of a “natural in” during consultations. At 3 months, 85% of trained HCPs who completed the follow up survey (*n* = 22) had shared learning with colleagues, indicating successful knowledge sharing.

**Conclusion:**

The one-hour education intervention appears to be an acceptable and feasible way to rapidly upskill time-pressured healthcare practitioners to discuss air pollution with patients. The “champion” model shows promise for wider dissemination, but widespread scale-up will likely require integration into medical curricula and clinical training pathways to ensure consistent, long-term education on air pollution.

## 1 Introduction

Air pollution remains one of the greatest environmental threats to human health worldwide. According to the Global Burden of Disease (GBD) 2021 study, air pollution was the leading Level 2 risk factor amongst all environmental and occupational risks, contributing to an estimated 236 million disability-adjusted life years (DALYs) and 8.08 million deaths globally in 2021 (1).

In the UK, air pollution is considered the largest environmental health risk (2), contributing to a wide range of chronic health conditions including cardiovascular disease, stroke, cancer and dementia (3). Long-term exposure can reduce life expectancy and carries significant costs to the NHS, estimated at £20 billion every year (4). On high pollution days there are more hospital admissions for heart and lung diseases and an increased risk of asthma attacks (5).

The 2020 inquest into the death of nine-year old Ella Kissi-Debrah whose hospital admittance and asthma attacks aligned with spikes in air pollution marked the first time modern-era air pollution was officially recognised as a contributing cause of death in the UK (6). The ‘Prevention of Future Deaths’ report that followed called for more information about the impacts of air pollution to be made available to the public and found that the health risks of air pollution were not being sufficiently communicated by Health Care Professionals (HCPs) to patients and their families (7). In response to this report, the Department for Environment, Food and Rural Affairs (DEFRA) funded Global Action Plan (GAP) to develop an education intervention for HCPs aimed at improving awareness and communication of air pollution risks. General Practitioners (GPs) are highly trusted health messengers who routinely advise patients on modifiable behaviours such as smoking, diet, and exercise (8–10). However, there is limited evidence that air pollution avoidance is routinely discussed in consultations or covered in standard medical training at undergraduate or postgraduate levels.

To address this gap, in 2023 GAP, Imperial College London’s Environmental Research Group (ERG), and the UK Health Alliance on Climate Change (UKHACC) co-developed an education intervention for HCPs. The intervention included an online training workshop and complementary patient-facing resources (leaflets, posters, online materials). The training workshop aimed to increase HCPs’ knowledge of the health impacts of air pollution and enhance their confidence in communicating exposure reduction strategies to patients. Following the training, HCPs were encouraged to act as air quality “champions,” sharing information with both colleagues and patients.

The development and evaluation of this intervention was guided by the UK Medical Research Council Framework for Developing and Evaluating Complex Interventions (11) which emphasises the importance of articulating a programme theory, co-designing with stakeholders, and systematically testing feasibility before full implementation.

The underlying programme theory hypothesises that increasing HCPs’ knowledge and confidence about air pollution will increase the communication of air quality advice in clinical practise. This, in turn, is expected to improve patients’ awareness of air pollution risks and their adoption of exposure-reduction behaviours. The theory was refined through co-design with HCPs to support the development of a relevant, acceptable and feasible training intervention.

The mixed-methods feasibility study presented in this paper aimed to explore the acceptability and delivery of the intervention, and to assess changes in HCPs’ self-reported knowledge and confidence, and perceived barriers and facilitators to communicating air pollution as a health risk within their practises.

## 2 Methods

### 2.1 Intervention design

The aim of the training intervention was to increase HCPs’ knowledge and confidence to discuss air pollution with patients and act as clean air “champions” within their practises, cascading down what they had learned with colleagues and peers.

The intervention drew on a “champions” model whereby trained individuals act as trusted messengers and advocates who disseminate knowledge and encourage wider uptake of practises within their professional networks (12). A similar “green champions” model was used to disseminate sustainable practises across the NHS staff (13) whilst “community champions” were trained to disseminate public health awareness information to community groups during the Covid pandemic (12). In this intervention, HCPs were expected not only to apply the learning within patient consultations, but also to share resources and learning with colleagues in their practises and professional networks.

The content of the training was co-designed with HCPs and clean air experts from Global Action Plan. Two in-person co-design workshops were held in January 2022, involving healthcare professionals from primary care settings (*n* = 8 HCPs, 3 GAP staff). The workshops were used to refine the structure, tone, content and practical delivery of the training intervention and accompanying patient-facing resources. Participants provided feedback on the relevance and usability of the proposed materials, including how air pollution discussions could be integrated into routine consultations. Draft training materials and resources were subsequently revised in response to this feedback prior to the training workshops being delivered.

The learning objectives of the training were for HCPs (1) to gain increased understanding of the health impacts of indoor and outdoor air pollution and what patients can do to protect their health; (2) to understand how patients can avoid exposure and reduce contribution to indoor and outdoor air pollution, advising on potential behaviour changes; (3) to consider patient engagement opportunities at different stages of the patient pathway; and (4) understand how to become a clean air champion.

The training included the following sections:

- Overview and importance of air pollution: the international context of air pollution morbidity and mortality.
- The UK context: including the burden on the UK’s finances, workforce and NHS services.
- Common sources of indoor and outdoor air pollution.
- The ways that air pollution impacts human health, including an excerpt from an online talk delivered by Professor Sir Stephen Holgate.
- Which patient groups are most relevant to receive information on air pollution.
- Opportunities on the patient pathway for informing patients about air pollution: Before, during and after a GP consultation.
- What practical advice can we give patients—reducing exposure and minimising contributions to both indoor and outdoor sources of air pollution.
- Resources available to help in the conversations with patients, including leaflets and posters featuring information on indoor and outdoor air pollution.
- How to become a ‘Clean Air Champion’—sharing the learning with colleagues and patients.
- Questions and discussion.

It was delivered to HCPs through an interactive one-hour online workshop, presented by a qualified medical doctor and air pollution specialist from the charity GAP. A one-hour workshop was deemed acceptable to HCPs during the co-design workshops, allowing sufficient content to be shared whilst recognising HCPs limited time availability. Alongside the training, HCPs were sent printed and electronic communications materials including leaflets, training slides, a teaching video, and suggested text for messaging and social media to support them in sharing their learning with colleagues and patients (training resources are available at http://www.actionforcleanair.org.uk/evidence-resources and the training slides are available at Clean Air Knowledge Hub for the Health Sector and attached as Supplementary File 1).

### 2.2 Theory of change and outcomes measured

**Figure 1:**
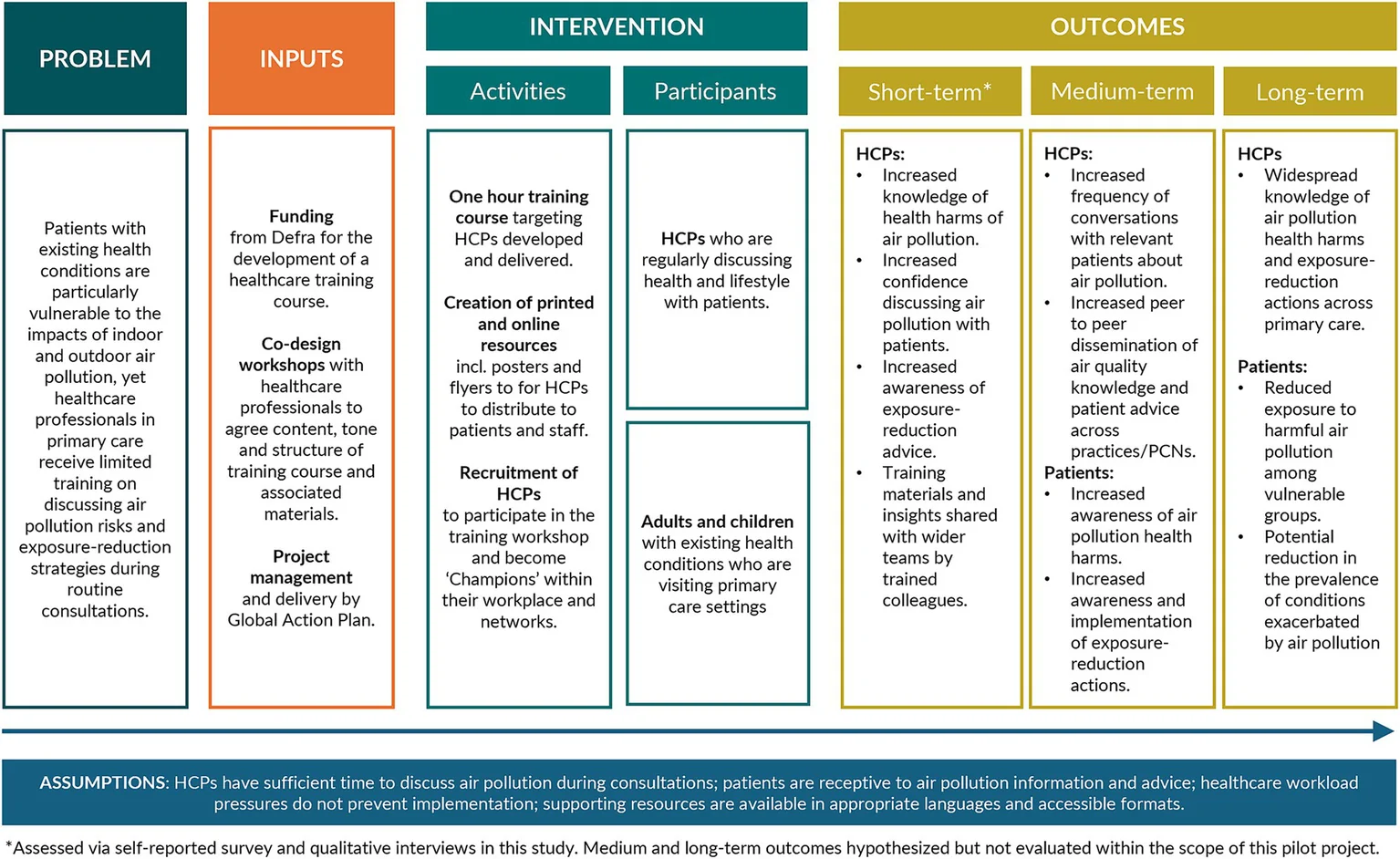
Theory of change and logic model illustrating the hypothesised pathways linking the air pollution training intervention for healthcare professionals (HCPs) with HCP, patient, and long-term public health outcomes. The model outlines the intervention inputs, activities, participant groups, and intended short-, medium-, and long-term outcomes. Short-term outcomes represent practitioner-level outcomes assessed within this feasibility evaluation through self-reported survey and qualitative interview data, whilst medium- and long-term outcomes represent hypothesised downstream impacts that were not directly evaluated within this study.

The intervention was underpinned by a Theory of Change (Figure 1) that depicts how training HCPs could lead to improvements in self-reported knowledge, confidence and ultimately patient awareness of and adoption of exposure reduction behaviours in relation to air pollution. This guided both the design of the intervention and the selection of outcomes for the feasibility evaluation.

In line with the UK Medical Research Council Framework for Developing and Evaluating Complex Interventions, the feasibility evaluation was informed by programme theory articulated through a Theory of Change (11). Theory-informed approaches are considered particularly important for complex public health interventions where outcomes are shaped by behavioural, organisational and contextual factors (11). The Theory of Change articulated the hypothesised pathways through which training healthcare professionals (HCPs) could improve their knowledge, confidence and communication practises relating to air pollution, and how these may subsequently contribute to increased patient awareness and adoption of exposure-reduction behaviours. It informed both the design of the intervention and the selection of evaluation outcomes.

The pathways were informed by evidence suggesting that improvements in trusted messengers’ knowledge and confidence are important precursors to behaviour change (14, 15). The evaluation therefore focused on assessing the acceptability of the training intervention to healthcare practitioners; the potential for the training to improve self-reported knowledge and confidence; the feasibility of using a ‘champions model’ to cascade knowledge from those who attended training to other HCPs and patients after the training. The outcomes used to assess each of these maps onto the Theory of Change (Figure 1) and are described in Table 1.

**Table 1:** Overview of the methods and indicators used to measure the outcomes of the clean air training intervention.

Domain	Indicators	How measured
Practitioner acceptability of the intervention	Usefulness of the training intervention and resources	Post-training and follow up surveys, interviews
Knowledge and confidence	Self-reported knowledge and confidence discussing air pollution with patients. Understanding of air pollution health impacts Understanding of what patients can do to protect their health Confidence talking to patients about air pollution Confidence giving advice to patients to reduce their exposure to air pollution.	Pre- and post-training surveys, interviews
Application in practise	Frequency of patient discussions, perceived patient receptivity	Interviews only
Use of resources	Usefulness and preferences (paper/digital) examples of use	Follow-up survey, interviews
Champions model	Extent to which HCPs shared learnings with colleagues	Follow-up survey, interviews
Integration/scaling up into HCP training pathways	Suggestions for embedding training in CPD or educational pathways	Interviews only

### 2.3 Study design

This study used a mixed-methods approach to assess the acceptability and potential impact of the online air quality training intervention for HCPs and to gauge its feasibility for use at a wider scale. The study combined pre- and post-training surveys, a 3 month follow up survey and semi-structured interviews with a sub-set of participants to explore short-term outcomes and experiences when implementing learnings into clinical practise (Survey questionnaires and interview topic guide are available in the Supplementary File 2).

In addition to assessing HCPs’ self-reported knowledge and confidence to communicate about air pollution, feedback was gathered to inform the refinement of the training intervention and to identify key barriers and enablers to putting the training into practise at scale.

#### 2.3.1 Participants and recruitment

Healthcare practitioners were recruited via communications shared by the Royal College of General Practitioners, Primary Care Respiratory Society, British Medical Association, UK Health Security Agency and networks including ‘Greener Practise’ and ‘Deep End’. Whilst the intervention was primarily targeted at GPs, allied professionals including nurses and pharmacists were also deemed eligible to participate given they also have face to face conversations with individuals who are vulnerable to the impacts of air pollution. Interested individuals registered online. To ensure the GPs that received training were representative of the population the socioeconomic distribution of patient groups was assessed by entering the postcodes of GP Champion practises into the government’s ‘English Indices of Deprivation’ website. The output showed a full range of scores, from 1 to 10, for both the ‘Income Decile’ and the ‘Multiple Deprivation Decile’ indices. Since 2015, GP practises have been able to register new patients who live outside the practise area, however we anticipate that a majority of patients reside within the area of their local GP practise. This being the case, the patient group covered by the study experiences the full range of income and deprivation levels.

#### 2.3.2 Data collection

Anonymous online surveys were distributed amongst those participating in the online education intervention before and after the training session. The purpose of the surveys was to assess the impact of the intervention on HCPs’ self-reported knowledge of air pollution and their confidence in discussing air pollution as a health risk with patients.

Three months after the training session, attendees were invited to complete a survey to assess the impact that the training had on their self-reported air quality knowledge, as well as to understand the extent to which course attendees were able to share their learnings with colleagues and patients. All surveys were hosted and distributed via the Qualtrics survey platform.

Following the 3 month follow up survey, training attendees were invited to register for a semi-structured interview to share their insights on the main barriers and facilitators they encountered when communicating air pollution as a health risk to their patients. Themes included GPs’ views on the value of air pollution advice; the information patients found most useful or wanted more of; how well GPs felt they delivered the advice; and its perceived impact on patients’ knowledge and behaviour. Ten interviews were carried out by MW with the support of a Global Action Plan team member. The interviews lasted 30 min on average and with the informed consent of the participants the interviews were audio recorded.

#### 2.3.3 Data analysis

Quantitative data from pre- and post-training surveys were analysed using Stata version 18 (16). For each of the four quantitative outcome indicators relating to changes in HCP knowledge and confidence (self-reported understanding of the health impacts of air pollution; understanding of actions patients can take; confidence discussing air pollution with patients; and confidence in advising patients) median and interquartile ranges (IQR) were calculated for both pre- and post-training responses. Mann–Whitney U tests were conducted to assess whether differences in pre- and post-training scores were statistically significant.

Qualitative data from the interviews were imported to NVivo (version 12) for thematic analysis (categorising respondents accounts in ways that can be summarised). All transcripts were read and systematically coded in full following a deductive coding approach, starting with a predefined set of codes to then construct larger themes.

## 3 Results

### 3.1 Quantitative results

Training was completed by 43 HCPs in total. They included 40 GPs, an advanced nurse practitioner, a pharmacist and a practise manager. All participants completed the pre-training survey (*N* = 43, 100% response rate), 40 completed the post-training survey (90%) and 26 completed the follow up survey 3 months after training had been completed (53%).

Over 60% of GPs surveyed reported that the training session was either “extremely useful” or “very useful” in preparing them to speak to colleagues about air pollution with 85% of those who completed the three-month follow up survey having shared the training with their colleagues. Additionally, 35% found it “moderately useful” and 4% as “slightly useful.” A similar proportion of attendees felt the training was either “extremely useful” or “very useful” in helping them talk to patients about air pollution, whilst 35% found it “moderately useful” and 8% “slightly useful.” At three months, 85% of respondents have shared learning with colleagues.

The training intervention led to improvements across all four self-reported knowledge and confidence outcomes related to air pollution (Table 2). Participants reported greater understanding of both the health impacts of air pollution and protective actions for patients, as well as increased confidence in discussing and advising on air pollution exposure (Table 2).

**Table 2:** Pre–post comparison of self-reported outcomes.

Outcome indicator	Median (IQR) Pre (*n* = 43)	Median (IQR) Post (*n* = 40)	*p*-value
Understanding of air pollution health impacts	7 (6–8)	8 (7–9)	z = 3.85, *p* < 0.001
Understanding of what patients can do to protect health	5 (4–7)	8 (7–9)	z = 5.25, *p* < 0.0001
Confidence talking to patients about air pollution	5 (4–7)	7 (6–8)	z = 4.01, *p* < 0.001
Confidence giving advice to patients to reduce their exposure	4 (3–6)	7 (7–8)	z = 5.53, *p* < 0.0001

Data presented as median (interquartile range). Respondents provided scores on a Likert scale from 1 (low) to 10 (high). Between-group differences were assessed using Mann–Whitney U tests. *p*-values < 0.05 were considered statistically significant.

The large majority (73%) of surveyed GPs reported finding the communications materials either “extremely useful “or “very useful” in helping them share air pollution information with their patients. An additional 19% found them “moderately useful,” whilst 4% rated them as “slightly useful” and 4% as “not useful at all.” Nearly 10% of the surveyed GPs reported to have perceived their patients as “extremely receptive” or “very receptive” when it came to learning about air pollution and ways in which they could reduce exposure to harmful pollutants. Whilst over 40% perceived their patients as “moderately receptive” and 38% as “slightly receptive,” 12% felt that their patients were “not at all receptive.”

Over 65% of the surveyed GPs reported barriers which prevented them from sharing information about air pollution with their patients. The consensus amongst all GPs surveyed was that “time” was their main barrier.

### 3.2 Qualitative thematic synthesis

Ten interviews were conducted with a subset of GPs who completed the training. Thematic analysis identified six domains: (1) perceived usefulness, (2) communication challenges, (3) patient receptivity, (4) practical educational needs, (5) structural/system barriers, and (6) enablers and strategies. A seventh domain summarises recommendations for future training and scale-up. Representative quotations illustrating each theme are presented below, with further supporting quotations available in Supplementary File 3.

#### 3.2.1 Perceive usefulness

GPs generally viewed the training positively and described it as both practical and relevant to their clinical role. A key benefit was that the training provided a clear structure for initiating conversations about air-quality with patients, helping GPs translate knowledge into actionable advice during consultations.

*“Overall, I thought the training was excellent and what I could put into practise from the training was really, sensible. It gave me a really good framework for being able to talk to patients about air quality” GP_3.*

In addition to supporting patient-facing discussions, GPs reported that the training increased their confidence to engage colleagues and initiate conversations about air quality within their practises. This suggests that the training may have potential to support wider organisational awareness and encourage the integration of air-quality considerations into routine primary care practise.

*“I feel a bit more confident knowing what to say, not just to the patients, but it was mainly initially to other members of staff, and to try and get things started in my practise” GP_6.*

#### 3.2.2 Communication challenges

The consensus amongst all GPs surveyed and interviewed was that “time” was their main constraint (GP consultations in the UK are generally 10 min in length).

*“Definitely time. You only have 10 min per consultation. Covering what they want in those 10 min is impossible anyway that is without bringing my agendas in there” GP_4.*

Several GPs highlighted that it was easier to initiate conversations on air pollution with patients that have certain health conditions such as COPD, asthma, or with parents of young children as they were more receptive to receiving information and advice on how to reduce exposure to harmful pollutants.

*“I found it was much easier to talk about air pollution when it appeared at face value to be moved directly related to what the patient had come in with. So, if I was speaking to a patient whose COPD wasn’t well controlled, I could talk about air pollution, similarly with asthma. Or maybe somebody with pregnancy or a baby cheque, it seemed very relevant to a small baby with developing lungs. But if somebody, I do not know, coming with knee pain or something random, it would be much harder to bring it into the conversation…” GP_9.*

Some GPs reported to have found more challenging to initiate conversation when there is not a *“natural in.”*

*“If somebody came in and there wasn’t a sort of a ‘natural in’ to air quality […] it was more difficult, because you do not want to sort of have a consultation on condition X, and then you just go, I’m going to talk to you about condition B. They’re like, but that’s not why I’m here. So sometimes I think that it’s about how to bring it into the conversations where there’s almost not a natural in” GP_3.*

Several GPs also reported to have found challenging to initiate conversations to encourage changes in behaviours, particularly when it came to promoting changes in lifestyles which are linked to people’s own contribution to air pollution.

*“Some people seem very like tied to cars and get defensive if you try and criticise anything to do with cars. So, I found that was also generally a little bit of an issue, but I found that if people were giving me more of the defensive, I just changed the topic. In general, I did not have any confrontation or discussions because you can kind of just sense of if it’s going to become really confrontational” GP_7.*

#### 3.2.3 Patient receptivity

Most GPs reported that patients who were more receptive to receiving information on air pollution were particularly interested in receiving advice and information about what they could do to reduce exposure to harmful pollutants and own contribution to the problem in their day to day lives.

*“I was surprised actually as to how receptive people were to hear about it, and I think they found it helpful knowing what they could do to reduce their exposure” GP_9.*

It was also highlighted that the messaging and information conveyed to patients really depends on what the patients’ particular circumstances are (e.g., damp/mould, proximity to busy roads, temporary housing) as these will ultimately determine the extent to which they could or not reduce their exposure to harmful pollutants.

*“I found that sometimes it kind of ended up with almost a feeling of a patient being locked into a situation they could not really do very much about. For example, living in a house that potentially has loads of issues with mould, but they cannot get it sorted because they have no power to do any of this stuff, despite the fact they are trying to raise complaints and they are living on the side of a really busy road. But also, they have been in like temporary housing for years […] these things that are just outside of people’s control. Almost felt like telling someone something they cannot do nothing about to any massive significant degree. But again, I think that’s where the whole communication thing might have been useful. I’m sure there are ways to maybe get around that, and maybe try and focus more on the things that people can do something about” GP_7.*

In general, most GPs highlighted that the information conveyed has been well received by their patients, but that there was limited evidence on the impact that the approach has had on patients’ behaviours. Some GPs suggested that to evaluate this it would be necessarily to engage with the patients directly, indicating the need for future research to explore patient perspectives and experiences.

*“I think they were quite receptive. They nod their head, they agree. Whether they will implement it practically or not is something we cannot monitor. Passing on the information, and making them aware I think is being done, but the next step I do not know how to do it” GP_8.*

#### 3.2.4 Practical educational needs

Although most GPs interviewed found the training sessions very useful, the large majority of participants highlighted that the training should have incorporated examples on how to talk to patients perhaps in a more interactive way (e.g., role play).

*“I think I would have liked to have had the opportunity, perhaps to practise or share ideas for actually how to have conversations with patients. I think it’s just Medicine in general. You can have the knowledge, but that does not necessarily mean you have got the skills or the sort of lingo to use when you are actually explaining it” GP_9.*

Some of the interviewed GPs suggested to have a stock of bespoke introductory sentences they could use when introducing the air pollution topic during different types of consultations tailored for different patients’ groups. It was also highlighted that the concise and visually engaging nature of the materials made them very useful. It was also noted that the materials should be made available in other languages to engage a wide range of groups, particularly those for whom English is not a first language.

Mixed views were expressed regarding the use of electronic and paper leaflets, those advocating for electronic resources highlighted that printed resources generate waste and people rarely referred to them. Supporters of printed resources expressed the view that some older adults and those who do not have access to smart phones or computers could benefit from having the resources in a printed form. It was also stated that having printed resources available in waiting rooms could spark patient’s interest in the subject and a desire to know more. The consensus, however, was that both type of resources are equally useful and valuable and that it would be up to the GP to decide which of these materials they find more appropriate for their own practise. Having the printed or online resources handy and ready to use could help them share the information even when there was not opportunity to mention it during the consultation.

*“I did not do anything until I had*—*or very little*—*until I had access to the leaflets. So, when I had the leaflets, it was much easier, because then I had something to share with them, and you use that as a framework” GP_4.*

Most GPs felt that whilst the training effectively highlighted the importance of discussing the issue of air pollution with patients and provided general information on the topic, additional learning and skills could only be developed through practical experience.

*“I think the training made me feel something I ought to do, but it did not really prepare me well for actually doing it. I think that when it came to actually doing it, it was very much learning. Learning whilst doing so. I sort of tried talking about it, see how receptive or not the patient was and sort of go from there, and then maybe try slightly different approach next time so it would be iterative, really sort of learning from experience” GP_9.*

GPs also highlighted that training should be incorporated early on during undergraduate medical school curriculums with continuous training updates that could serve as reminders.

*“I think it is really useful for trainees because we are already changing the way that we consult or like practising the way that we consult and building things in and getting a kind of structure. I think it’s useful to have things like this built in from the beginning and to have an awareness right from when you start practicing” GP_6.*

#### 3.2.5 Structural and system barriers

GPs highlighted that there were limited opportunities to incorporate air pollution as an addition to current standard consultations and that therefore, occasions should be identified, where the topic could be included automatically as part of existing consultations protocols.

*“I’ve been saying, you know that there’s no room for it being over and above normal work. It just has to be included as part of the normal stuff that we talk about with patients like smoking. I suppose you just have to treat it like smoking really in many ways” GP_2.*

*“If we are really seriously going to get this used, it has to be incorporated into protocols, because clinicians are just so busy and we do not have time to do another thing. We just forget and so our plan*—*and I do not obviously do not know if it’s going to work yet*—*but this should be included it in the processes for the nursing team and the physician associates, who are doing long term conditions, because actually those are the patients that are going to benefit the most, so that it’s automatically a part of the treatment of their long term condition” GP_5.*

GPs emphasised that air-pollution advice competes with crowded clinical agendas unless it is built into routine pathways. Several interviewees argued for integrating air-quality content into existing long-term condition reviews, so that it becomes a default rather than an add-on, for example within respiratory education and care plans for asthma and COPD.

*“I think that if we are having any training on asthma and COPD, air quality and air pollution should be included in that. So that then it just becomes a kind of standard education” GP_6.*

#### 3.2.6 Enablers and strategies

##### 3.2.6.1 Communication tactics

Some GPs reported to have adopted a “positive message framing approach” when communicating information about air pollution to their patients, focusing on the things that people could do something about.

*“I think the patients that we really need to be having conversations with are those who have got lots of chronic conditions. Those that are already affected by air pollution and have lots of different modifiable risk factors. We need to be talking to them about this, but these patients are potentially much more resistant to talking. You know, they already feel like they are being told to change lots of things and so this is a kind of another thing that I feel like I’ve been told to change. So, I think with them it’s much more about trying to be positive and to talk about the benefits and things that they could do,” GP_6.*

Some GPs highlighted that bringing the air pollution issue to a “local level” was a very good way for engaging with patients.

*“I think go as local as possible. Go down to your postcode, your workplace. This is you. This is what you are breathing in and it’s not what you are breathing in 2030 or 2050. It’s what you are breathing in now, today”GP_3.*

Others mentioned that having their own bikes in the consulting room has served as a “prompt “for engaging with patients and initiating conversations on active transport, health benefits and air pollution exposure reduction and own contribution.

*“Having my electric bike in my consulting room creates a lot of opportunities for discussions as well. You know, simple prompts that can actually get people talking are really useful” GP_1.*

It was also highlighted by some GPs that they could indirectly promote healthier cleaner environments by weaving environmental advice into discussions about managing other health conditions. This suggests that air-quality advice may be most acceptable and meaningful to patients when framed within their wider health needs rather than as a standalone issue.

*“This particular person, had many problems and, one of the things we talked about was, maybe making sure she was going outside and getting some exercise. But making sure she did that in a place that was green and we talked about some local places where she lived near that she might go and how she might get there just for the air benefit, for her mental health, with all those kind of things and she was open to that” GP_6.*

Some GPs highlighted that having resources such as those developed by GAP in a “ready to use” form was extremely useful as it saves time and facilitates the sharing of information with others.

*“I think the provision of the slides and the resources is fantastic because they just, you know, writing presentations takes a long time. So, like the presentation I gave, I felt very confident. I just use those slides that I’d seen before. So, I think giving people the resources to then use them to educate others” GP_4.*

##### 3.2.6.2 Champion model

Following the training, many GPs described adopting the “clean-air champion” role, cascading learning across their practises and wider primary-care networks. Champions delivered talks to clinical teams, coordinated Clean Air Day activities, and circulated materials through newsletters and shared resources through GPs WhatsApp groups and Clinicians messaging groups. Other GPs mentioned to have use part of the resources as starting point for own resources and or presentations delivered within their practise.

*“Use pre-existing networks (e.g., Greener practise local groups, training hubs) that can reach a broader range of the primary care team. Also, really important to include nurses especially as they do most of the asthma and COPD reviews. Also, should include the wider primary care team (pharmacists, HCA, admin etc) so that everyone is aware and can act together, e.g., altering staff travel patterns/being role models etc” GP_7.*

*“I have used the slides but modified them to make them more personal to our practise” GP_4.*

Some GPs found that setting a positive example was a first step for initiating discussions about air pollution with colleagues and patients.

*“I’ve always felt that if everybody’s driving to work and the car park is full of cars, we cannot really expect patients to walk to the surgery. I think it sets a really bad example basically. That was a lot of what I was trying to do. Education is more about linking together what we know about air pollution, its effects, what we are doing and the message that sends to patients. Linking that all together” GP_6.*

#### 3.2.7 Recommendations for future training and scale up

##### 3.2.7.1 Audience and channels

GPs highlighted that consistent, multi-channel messaging increases salience and legitimacy, making conversations easier and uptake more likely. Reinforcement from external authorities (e.g., CCGs, local/national campaigns) was seen as particularly helpful.

*“I think hearing it from different […] sources, I think that is maybe what makes the difference. I noticed that we had an announcement about Clean Air Day from our local CCG and that was amazing, it really felt like I was being backed up in a way, I did not have anything to do with that. It just came from a different sort of source. So, I think just public awareness campaigns locally or nationally make a big difference because it’s just the same message coming from different sources” GP_10.*

Specific health care professionals such as midwives, obstetricians and respiratory nursing teams were recognised as key players when endeavouring to raise patients’ awareness of air pollution. Several GPs highlighted that official training on air pollution should be offered to health care professionals through the Royal College of Physicians. Other training avenues suggested included NB medical, primary care respiratory society, Society of Medicine, Fourteen Fish appraisal tool kit, Clinical Knowledge Summaries (CKS), GP Notebook, Blue stream (mandatory training) and through nursing update or the physician associate update.

##### 3.2.7.2 Content framing

During the interviews GPs noted that whilst many health professionals are aware that poor air quality affects health, understanding of the scale of the health impacts, the range of populations affected, and the extent of the problem at a local level often remains limited. However, participants suggested that exposure to evidence and local data could increase awareness and engagement.

*“I think people (colleagues within the GP practise) were willing to hear about it. Some people did state the fact that, perhaps historically with regards to something like idling, for example, they would have been quite sceptical about the impact. But I think once people actually see that the facts and figures, people are astonished really that they have not perhaps learned about it before and agree that it is something that they want to talk to people about” GP_4.*

Some GPs suggested that air pollution should be presented as a “multi system pathology” enlightening that the whole body can be affected by air pollution not only the respiratory organs.

*“I think just the fact that GPs have not really made the connection between air pollution and their consultations and the whole body being affected rather than just respiratory. So, I think from an awareness raising point of view. I think we need to get over this common misconception that I’ve come across and that I had myself. That air pollution equals respiratory and get beyond that and somehow get it filtered into teaching sessions or Continuing Professional Development (CPD), whatever for everything, rather than just talking about it in a respiratory or asthma session” GP_2.*

It was also suggested that when disseminating air pollution information, the co-benefits of improving the quality of the air we breathe should be clearly conveyed and should be linked to the climate change agenda.

*“Highlighting some of the core benefits that actually you get, if you get people out of the car. They get, you know, an individual benefit from doing physical activity and disease reduction as a result […] and there’s also that sort of societal benefit and the planetary benefit. So actually, you can get person and practise, and population and planet all benefiting from the same action, and this is how we get that sort of co benefit” GP_3.*

## 4 Discussion

### 4.1 Summary of main findings

The evaluation provides important signals on the feasibility and acceptability of delivering clean air training to HCPs. Recruitment to the intervention was successful, survey response rates were high, and the training itself was well received with the majority of participants rating it as very or extremely useful in preparing them to speak with both patients and colleagues. Moreover, of those who completed the three-month follow up (*n* = 26), 85% reported that they shared the learning with others in their team. Qualitative interviews reinforced these findings with participants describing the training as relevant, motivating and a useful starting point for incorporating air pollution into clinical conversations. The provision of ready-to-use communications materials was viewed as particularly valuable in supporting introduction within time-limited conversations. Together these results suggest that a champion model is deliverable in primary care settings and acceptable to clinicians, whilst also highlighting areas such as sustaining engagement over time and embedding air pollution within standard care pathways that will need further development before progressing to a larger-scale effectiveness evaluation.

### 4.2 Champions model: barriers and enablers

Several factors enabled HCPs to act effectively as clean air champions. The training intervention improved self-reported knowledge and confidence, and many participants valued the provision of concise resources that could be shared with patients and colleagues. Conversations were easier to initiate when linked to relevant health conditions such as asthma, COPD, pregnancy or child health, and parents of young children were often particularly receptive. Positive, locally framed messages and simple prompts (such as visible cycling equipment in consulting rooms) served as useful conversation starters.

At the same time, participants identified significant barriers, particularly the constraints of 10-min consultations, the absence of a “natural opening” in some consultations, and concerns that discussions could feel futile, or raise anxiety, if patients had little control over their environment (e.g., housing conditions or proximity to busy roads). Some HCPs reported defensiveness from patients around topics such as car use, which made behaviour change conversations more challenging. A further limitation is that whilst information sharing was generally well received, we do not yet know the extent to which these conversations translate into sustained changes in patient behaviour – and issue that will require further exploration in a future effectiveness study.

### 4.3 Comparison with other champion models

During the COVID-19 pandemic, a community champion model was used to impart public health information to vulnerable populations through trusted figures such as faith leaders, volunteers and community workers (13). Unlike that model, where individuals were often recruited without formal training and reported that structured preparation would have been valuable, our study demonstrates that targeted training for HCPs can measurably increase self-reported knowledge and confidence in addressing air pollution with patients. Both models relied on trusted messengers embedded within their communities, but in our case, these were GPs and other clinicians whose professional credibility reinforced the message. As with COVID champions, participants emphasised that real competence to share air quality messages developed through practise, and that simple practical advice and supporting materials were critical. The COVID champion experience also highlighted challenges that resonated with our findings: the persistence of misinformation and mistrust, the need for more interactive communications skills training, and the difficulty of evidencing direct impacts on patient behaviour. When investigating how community health professionals can be empowered to communicate air pollution information and advice, Tan et al. (15) also reported similar findings, highlighting that there is potential for community health workers to be integrated into air pollution communication strategies, leveraging on their established connections and trust with the community.

During the interviews it was noted that whilst many health professionals are aware that poor air quality affects health, levels of understanding on how much harm poor air quality can cause or the different groups of people that could be affected, as well as on the extent of the problem at a local level remains low.

### 4.4 Lessons for future implementation

Participant feedback also highlighted several areas that should be strengthened in future iterations of air quality training programmes. Many participants expressed a desire for more opportunities to practise communication skills and apply learning to realistic clinical scenarios, suggesting that longer sessions and interactive approaches such as role play may improve confidence in translating knowledge into practise. Given the significant time pressures faced by healthcare professionals, flexible online learning modules that can be completed asynchronously may also improve accessibility and engagement.

Findings from this study further suggest that whilst GPs are important trusted messengers, other members of the wider primary care workforce including practise nurses, specialist nurses, pharmacists and health visitors may have greater opportunities to provide ongoing preventative advice and reinforce key messages with patients. Future programmes should therefore consider multidisciplinary delivery models and broader integration of air pollution and environmental health into healthcare education and professional curricula.

Participants also emphasised the importance of tailoring advice to patients’ social and environmental circumstances to avoid recommendations that may feel unrealistic or insensitive to structural constraints such as poor housing or limited transport options. Future training should therefore place greater emphasis on context-specific, patient-centred communication strategies. Whilst translated resources and wider patient engagement approaches may help improve accessibility and inclusivity, further work is needed to directly understand how patients receive, interpret and act upon air pollution advice from clinicians in practise.

Finally, whilst this intervention was developed within a UK primary care context and co-designed with UK healthcare professionals, several broader implementation lessons may be transferable to other healthcare settings internationally. In particular, the findings highlight the potential value of healthcare professionals as trusted messengers for communicating environmental health risks and preventative advice within routine care. The study also identifies common implementation challenges likely to be relevant across healthcare systems, including limited consultation time, the need for practical communication training, integration into existing care pathways, and the importance of multidisciplinary approaches to patient education. However, healthcare systems differ substantially in their organisation, workforce structures, consultation models and opportunities for patient interaction. Further research would therefore be needed to adapt and evaluate the intervention in other healthcare contexts before assuming transferability. Rather than providing a universally transferable intervention model, this feasibility study offers early implementation insights that may help inform the adaptation and development of environmental health training initiatives in other contexts.

### 4.5 Implications for practise and policy

The findings point towards practical avenues for embedding clean air conversations into routine healthcare appointments. In line with recommendations made by the Royal College of Physicians in 2016 (4), the latest Royal College of Physicians report (17) and the NHS 10 Year Health Plan (18), participants emphasised that air pollution advice should be normalised and embedded within existing care pathways, such as asthma or reviews, maternity care and child health visits. This could be supported by system-level innovations, such as alerts in GP records flagging patients in high-pollution postcodes as has been trialled at Great Ormond Street or tying in with high-pollution day notifications that trigger timely advice (19). Whilst time constraints remain a barrier, short prompts, leaflets or alerts could create “natural openings” for conversations. In addition, framing messages positively by emphasising the co-benefits may make advice more acceptable to patients. At a wider policy level, combining professional training with system supports and aligning with public health campaigns like Clean Air Day could amplify the impact by reinforcing messages across multiple trusted sources (20).

### 4.6 Limitations of the study

This evaluation has several limitations that should be considered when interpreting the findings. First, as a feasibility study it was not designed or powered to assess effectiveness, and outcomes relied on self-reported measures of knowledge, confidence and implementation rather than independent objective measurements of behaviour. As a result, reported improvements may not necessarily reflect actual changes in clinical practise or communication behaviours. The absence of a control group and the anonymous nature of the survey also limited our ability to attribute changes solely to the intervention or to assess changes at the individual participant level. Future studies could strengthen evaluation through the inclusion of linked before/after data, objective knowledge assessments (e.g., short quizzes) and behavioural outcome measures gathered directly from patients.

Second, the sample size was relatively small and participants were recruited voluntarily through professional and sustainability-focused networks, raising the possibility of selection bias towards HCPs who were already motivated or interested in environmental health issues. As recruitment occurred through professional networks and organisational communications rather than direct invitations to a defined sampling frame, it was not possible to determine the number of HCPs exposed to the recruitment materials, calculate response rates, or compare participants with non-participants. The extent of selection bias therefore cannot be quantified, and this may limit generalisability of findings to wider healthcare settings. In addition, follow-up response rates declined to just over half of original participants at 3 months, meaning those who remained engaged in the evaluation may also have been the most likely to apply or value the training, potentially leading to overestimation of acceptability and implementation success.

Third, whilst participants reported perceived improvements in communications practises and described patients as generally receptive to discussions about air pollution, these findings were based solely on HCP perspectives. The study did not include direct assessment of patient experiences, behaviour changes, or health outcomes and therefore conclusions can not be drawn regarding patient-level impact. Further research should incorporate patient perspectives and explore whether increased clinician confidence and communication translate into sustained changes in patient awareness, behaviour and exposure reduction practises over time.

Finally, the findings reflect a preliminary feasibility study within UK primary care settings and should therefore be interpreted primarily as early implementation insights, rather than evidence of implementation effectiveness. Despite these limitations, the study provides valuable insights into the feasibility, acceptability, and practical implementation considerations associated with using a clean air champion model in primary care settings.

## 5 Conclusion

In summary, this feasibility evaluation suggests that training HCPs to act as clean air champions is both acceptable and deliverable in primary care. The training intervention improved HCPs self-reported knowledge and confidence was viewed as useful in supporting conversations with patients and colleagues and provided resources that could be readily integrated into time-limited consultations. Whilst further work is needed to understand patient-level impacts, incorporate more robust behavioural and implementation outcomes measures, and to test the effectiveness of the champions model at scale, these findings indicate that primary care professionals can play a valuable role in communicating the health risks of air pollution and supporting patients to take protective action. Future implementation efforts should focus on embedding air pollution education within existing healthcare training pathways, incorporating more practical communication training, and broadening delivery across multidisciplinary healthcare teams. Embedding this approach within existing clinical education, supported by system-level tools and wider public health campaigns, would enhance its impact and scalability.

## Data Availability

The original contributions presented in the study are included in the article/Supplementary material, further inquiries can be directed to the corresponding author.
