# Supplementary material for: Exploring the feasibility and acceptability of an educational intervention on air pollution for healthcare professionals
Source: Front Public Health. 2026 Aug 12;14:1862924. doi: 10.3389/fpubh.2026.1862924 (PMC13537046; doi:10.3389/fpubh.2026.1862924)
Supplement: Supplementary file 1 [file Data_Sheet_1.PDF]

Clean Air Champions

Air Pollution + how to advise patients about it

201 Borough High Street, London, SE1 1JA, Telephone 0203 8177636
Charity registered in England and Wales No. 1026148, in Scotland No. SC041260, Registered company in England and Wales No. 2838296, VAT No. 625 994 009

- REMEMBER TO SELECT 'SHARE SOUND' IF DELIVERING ACROSS TEAMS/ZOOM, WHEN YOU SHARE YOUR SCREEN.

Agenda

Session Topics
• Talking to Patients about Air Pollution – Oversight and Importance
• Health Impacts of Air Pollution – Presentation with Sir Stephen Holgate
• Having Effective Conversations with Patients About Air Pollution
• Becoming a Clean Air Champion
• Winding up

Learning Objectives

- Gain increased understanding of the health impacts of air pollution and what patients can do to protect their health.
- Understand how patients can avoid exposure and reduce contribution to air pollution, advising on potential behaviour changes.
- Consider patient engagement at different stages of patient pathway & see leaflets
- Knowing how to become a Clean Air Champion.

Pollution hotspots

- Map example from Islington, London
- Main roads are common culprits

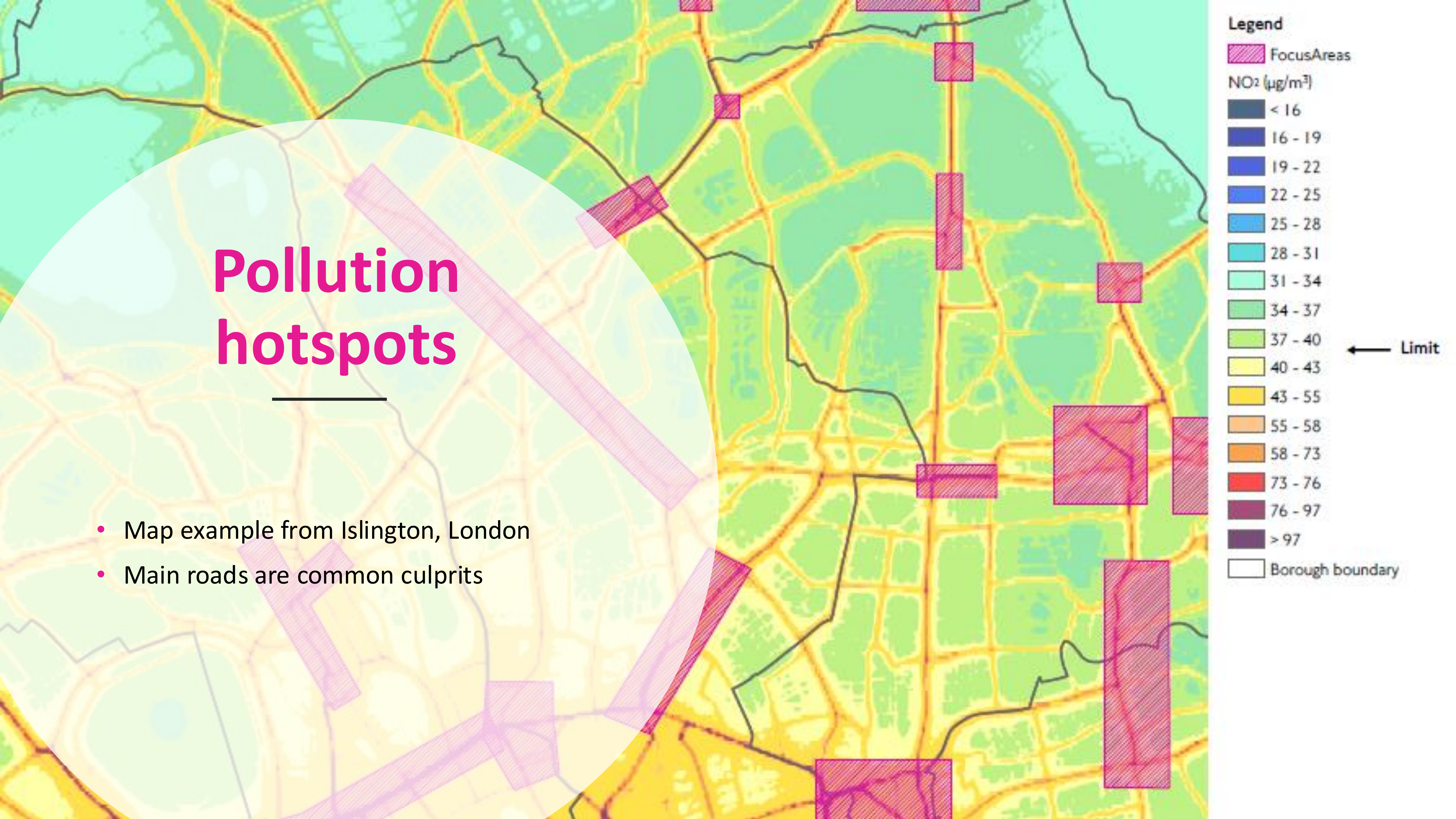

Talking to Patients about Air Pollution

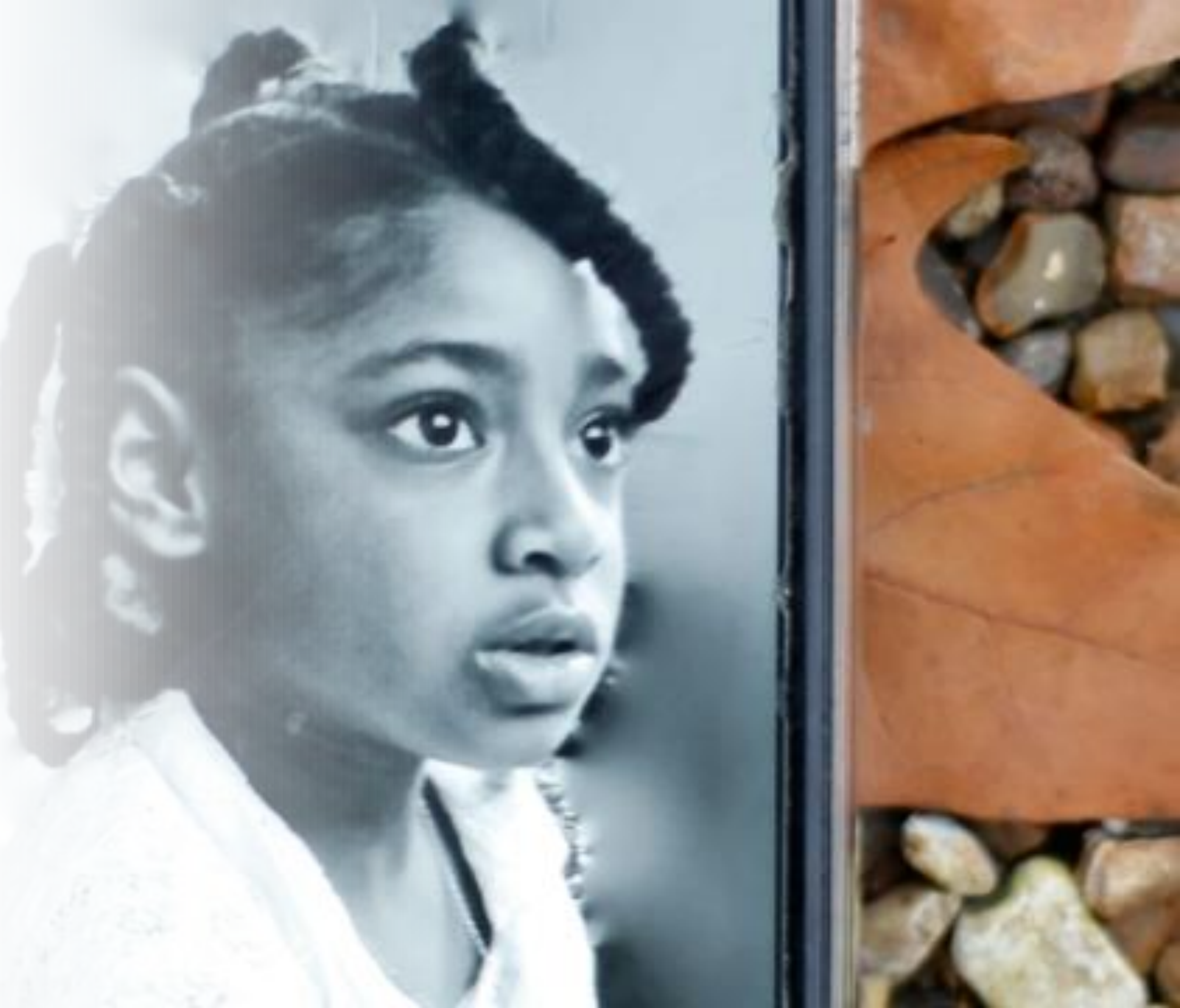

- Ella Kissi-Debra and Future Prevention of Death Report.
- Asthma Models of Care – primary care to act on air pollution.

Royal Colleges Round Table Discussions:

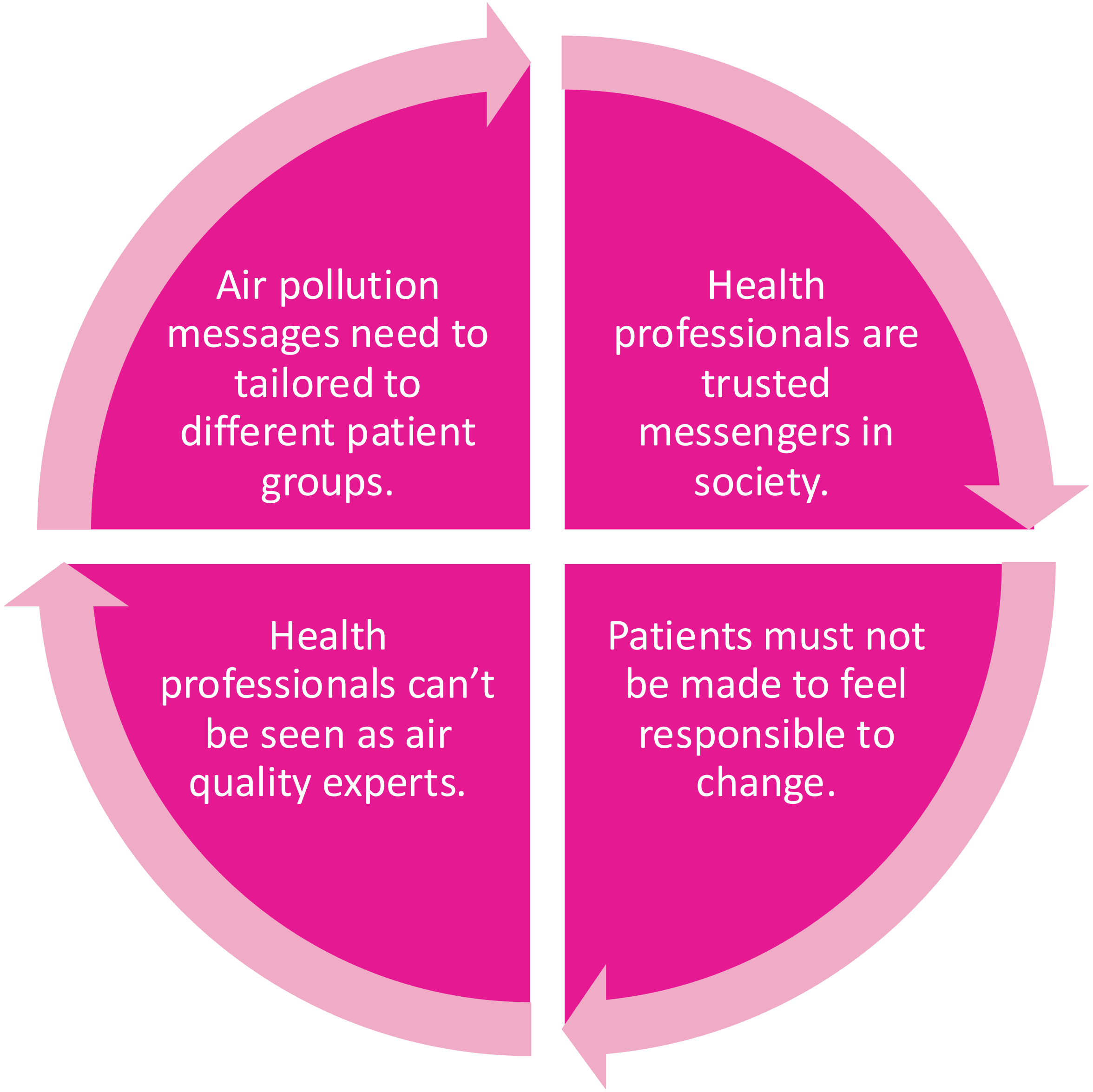

Sir Stephen Holgate

Video – The Health Impacts of Air Pollution

5m34s to approx 15m 30s

Air pollutant particles ($PM_{2.5}$ and $PM_{0.1}$) become systemically bio

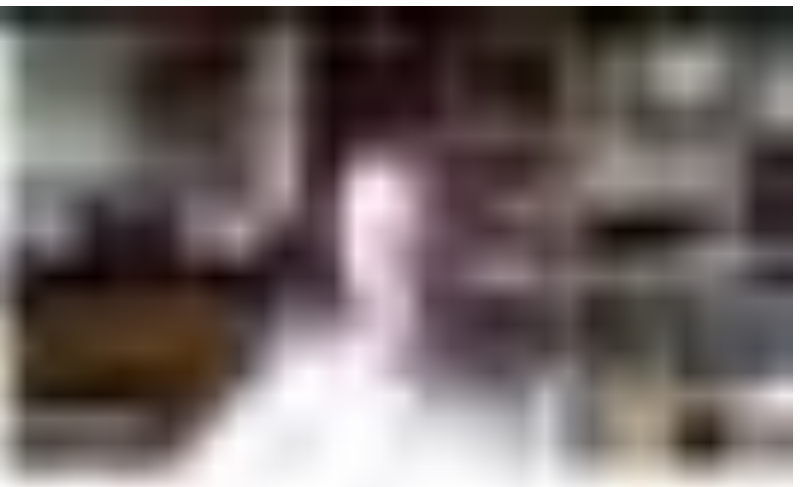

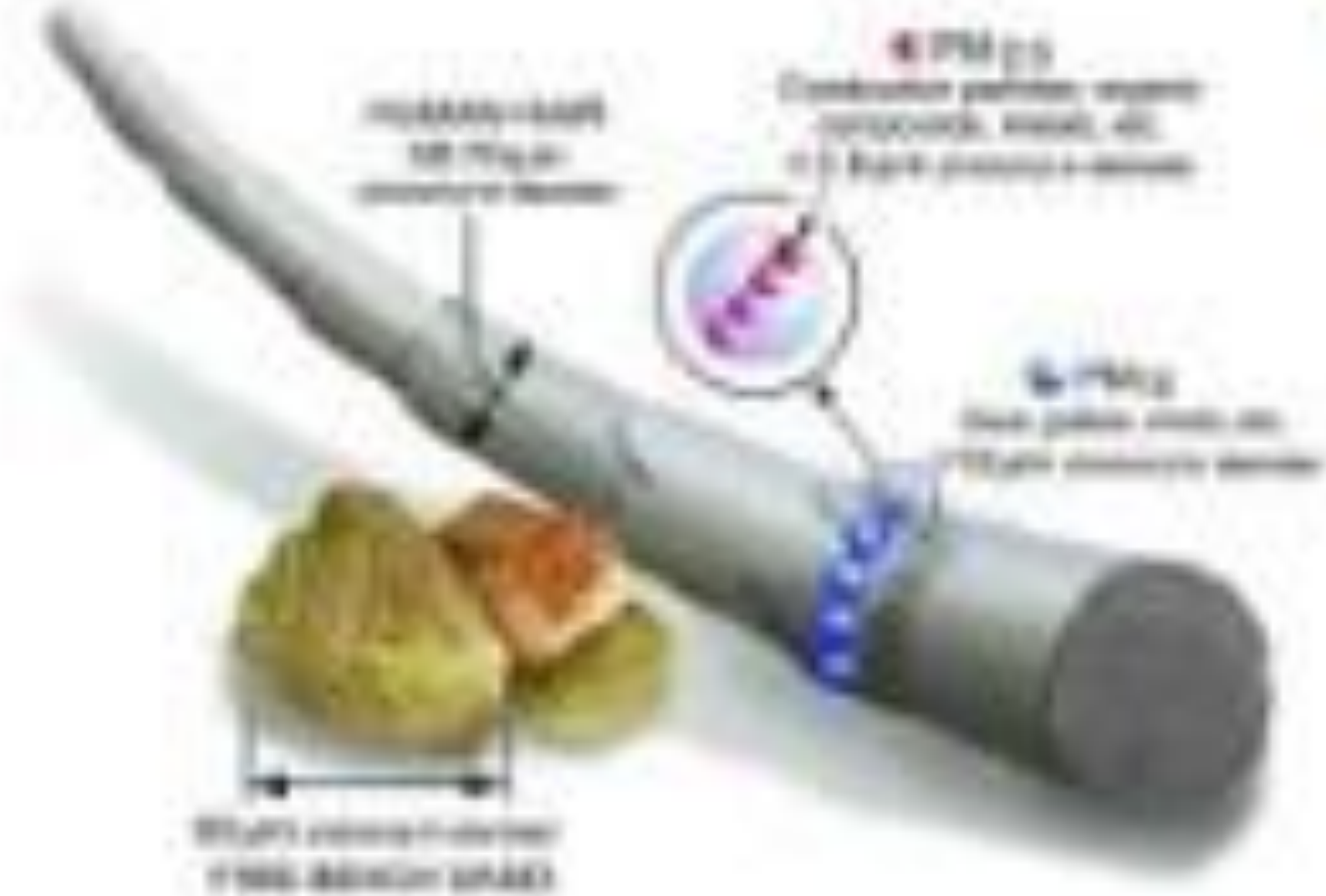

Pathologies --> Patient Groups

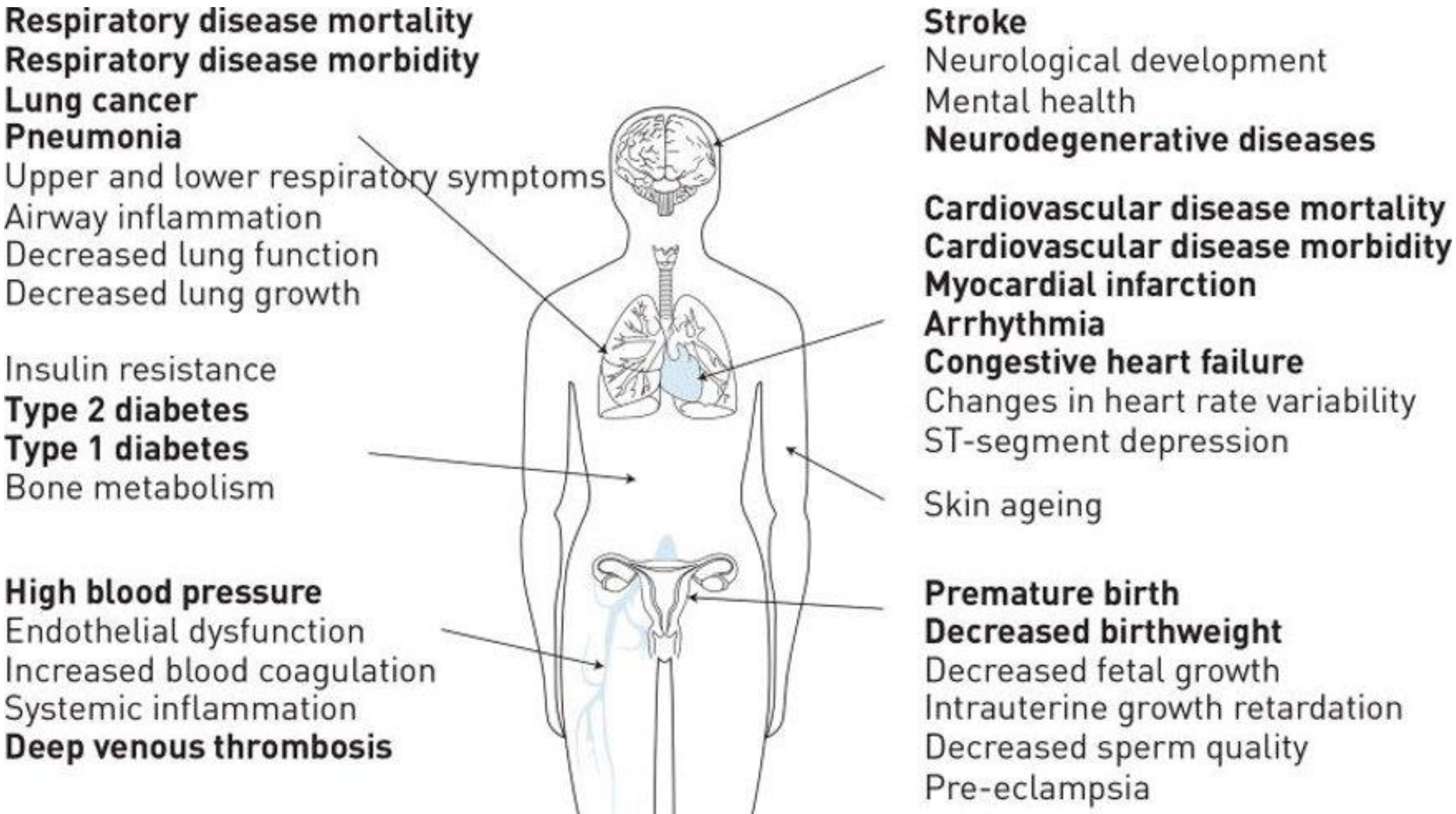

Patient Groups (non-exhaustive!)

- Asthma, - Child and Adult. Cystic fibrosis etc
- COPD & Emphysema, Bronchitis, Bronchiolitis. Fibrosis
- All Children - impacts on development!
- Pregnancy & Fertility
- Heart and Circulatory disease
- Diabetes
- Dementia
- Mental Health, e.g. Anxiety/Depression.
- Elderly people

Effective Communications

- Awareness and behavior change more likely with reinforced messages, e.g.
- Patient waiting area or phone message/website
- Consultation with health professional
- Leaflet or follow-up text/pdf/email

Pre-Consultation Opportunities

- In-person vs electronic consultations
- Website
- Phone
- Text messages
- Practice social media pages
- Appointment reminders
- Prescription requests
- Waiting room posters/screens/notice boards
- Patient registration forms
- Invitation letter to routine/regular reviews

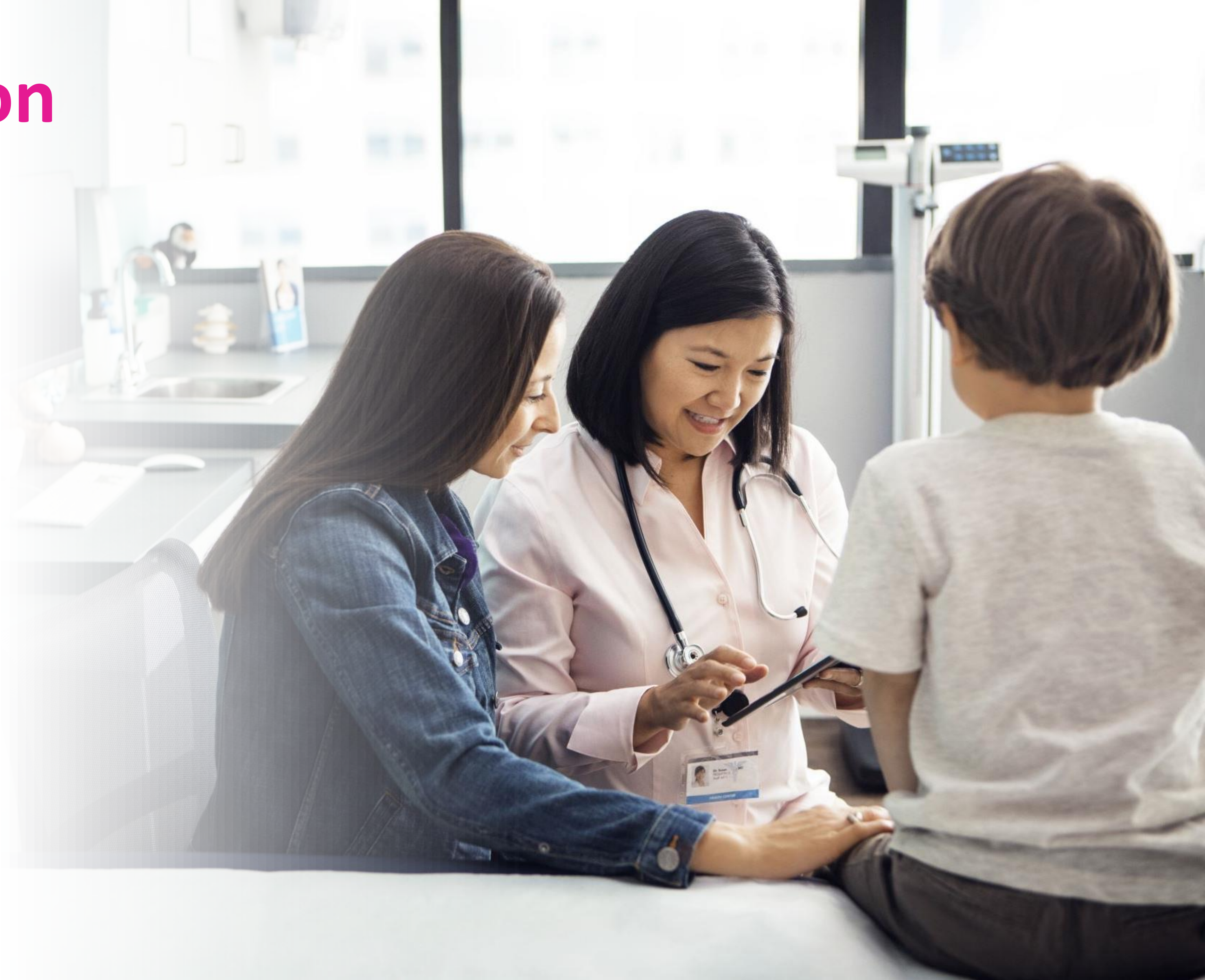

During Consultation

History	Integrating into conversation – e.g. "I'm breathless doctor"
Management plan	Specific, achievable goals
Lifestyle advice	Work through a leaflet together<br>If walking for exercise, try a green space
Closing Statements	Signposting

Post Consultation Opportunities

Link workers & Social Prescribing – Walking groups in green spaces/side streets

Posters in Pharmacies

Post Consultation Signposting

PDFs, emails and texts

Letters to patients

Specialist Consultations as Opportunities

Annual asthma review – personalised asthma plan	Annual 'health check-up'	Diabetes reviews
COPD reviews	48-hour post-discharge reviews	Midwife/pregnancy checks
8 week baby checks	Vaccine/flu jabs/childhood immunisations	Phlebotomy

Dr Sinead Millwood

What is the air quality in Manchester like doctor? - YouTube

1m 41s – 3m 53s

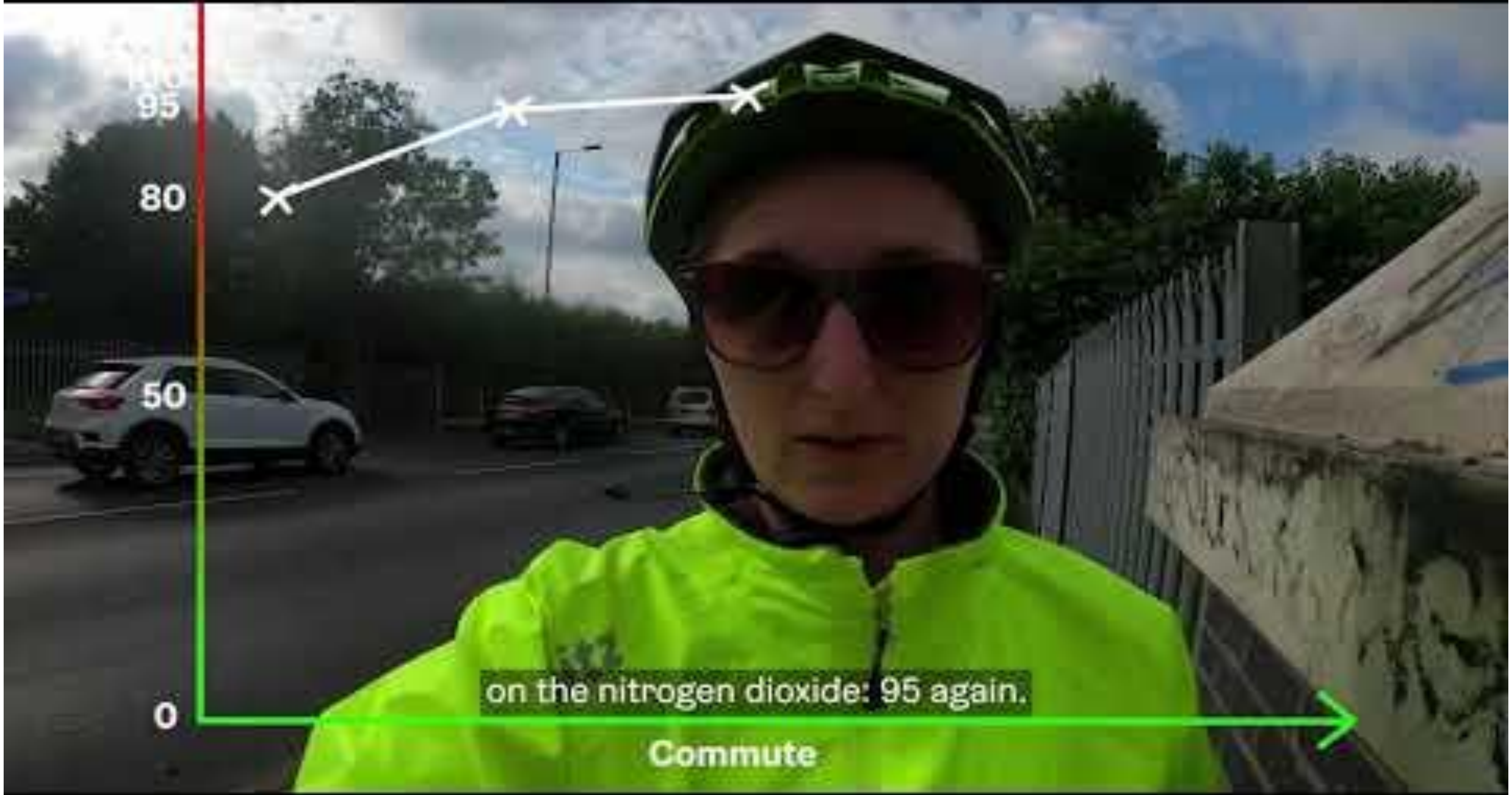
95
80
50
0
on the nitrogen dioxide: 95 again.
Commute

Patient Leaflets

- Patient Leaflets designed for a National GP project
- Applicable to secondary care too
- Specific messaging for patients

Air Pollution & Health

How does air pollution damage my health?

- Air pollution is generally invisible but affects our health from before birth through to old age. It enters our lungs when we breathe and gets into our blood, leading to effects throughout the body. There is no safe level of air pollution and there are many causes, inside and outside the home.

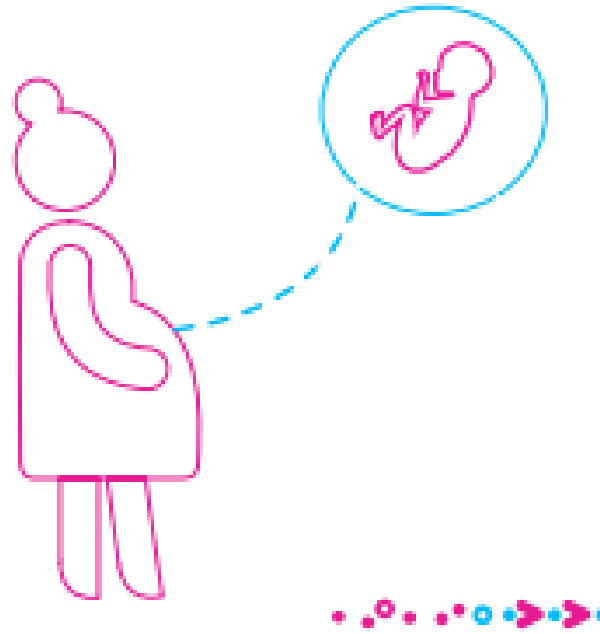

- We are all affected by air pollution whether we live in the town or countryside. It is generally worse near main roads because of traffic.

- Air pollution enters your bloodstream via your lungs. It causes and worsens heart and lung diseases and is linked to higher blood pressure and strokes. Long term exposure can increase your risk of lung cancer.

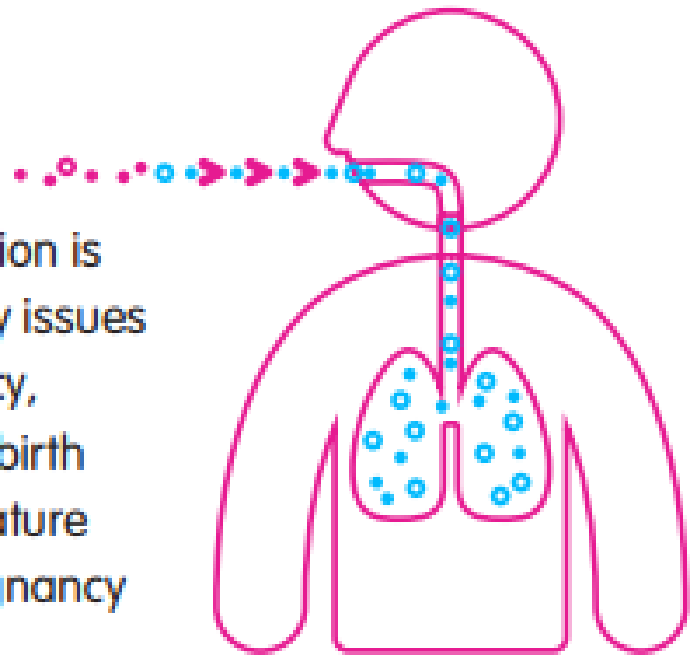

- High air pollution is linked to many issues with pregnancy, including low birth weight, premature birth and pregnancy loss.

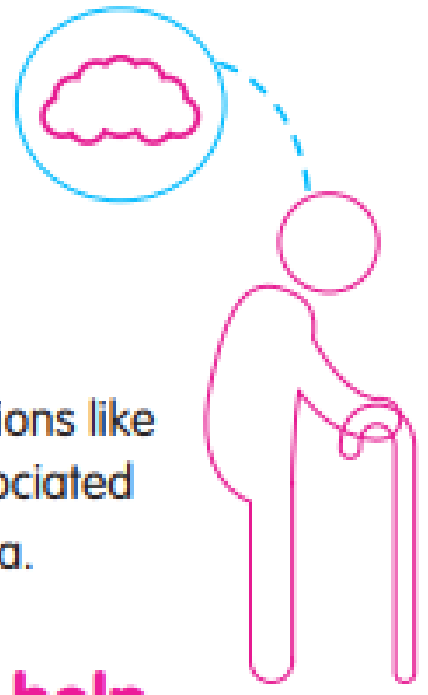

- Air pollution makes people more likely to suffer infections like bacterial pneumonia and possibly COVID 19. It is associated with mental health issues, memory loss and dementia.

But there are things we can all do to help

Find health expert approved guidance with simple steps that you can take to tackle air pollution and protect your health at

cleanairhub.org.uk

Simple steps can have a big impact on the air you and your family breathe

Make travel choices for cleaner air

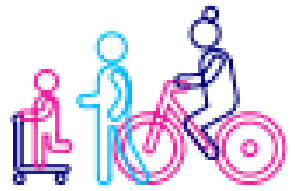

Use people power – Walk, scoot or cycle to work or school whenever you can.

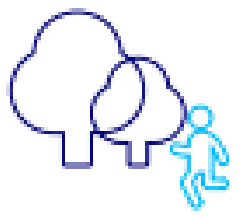

Discover the side streets – Use quieter streets when you're walking or on a bike to avoid the higher levels of air pollution on main roads.

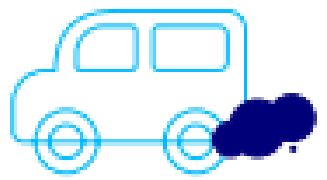

Don't idle – If you have to drive, turn off the engine when you are not moving and it is safe to do so. Consider switching to an electric vehicle. Air quality can be worse inside the vehicle than outside.

Make cleaner air decisions in the home

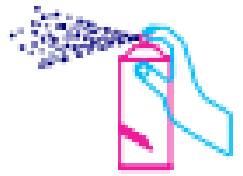

Use fragrance-free, milder cleaning products and avoid plug-in fragrances.

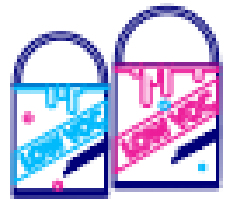

When decorating, choose safer paints and varnishes labelled 'low VOC' (volatile organic compounds).

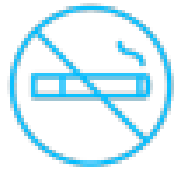

Ask people not to smoke in your home.

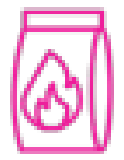

Reduce home burning as much as possible (e.g. log burners, coal fires or candles).

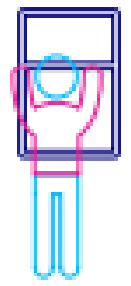

Ventilate your home - Open windows and use extractor fans when cooking or using cleaning products, but close windows near busy roads during rush hours.

global action plan
Department for Environment Food & Rural Affairs

Air Pollution & Child Health

How does air pollution damage your child's health?

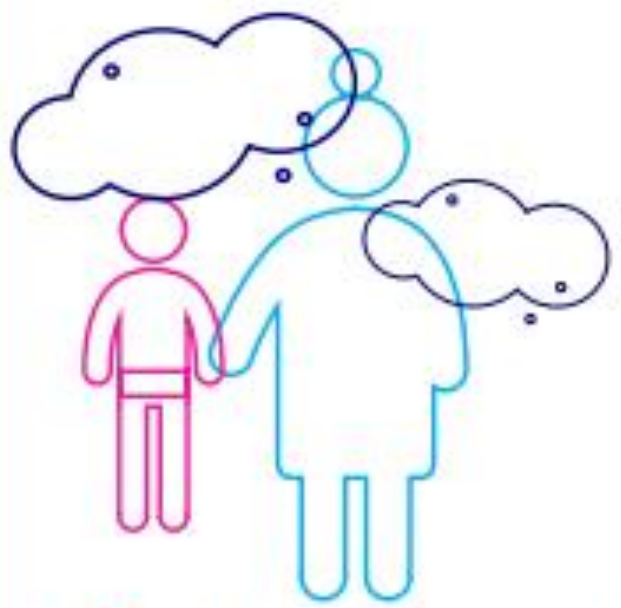

- Air pollution is generally invisible but affects our health from before birth right through to old age. It enters our lungs when we breathe and gets into our blood, leading to effects throughout the body. There is no safe level of air pollution and there are many causes, inside and outside the home.

- Children are especially sensitive to dirty air because their bodies are still growing. Their lungs, immune systems and brains are impacted by air pollution, and it also affects their ability to learn.

- We are all affected by air pollution whether we live in the town or countryside. It is generally worse near main roads because of traffic and, because children are small, they often are closer to sources of air pollution such as car exhausts.

- Air pollution can play a part in causing asthma and can make symptoms worse.

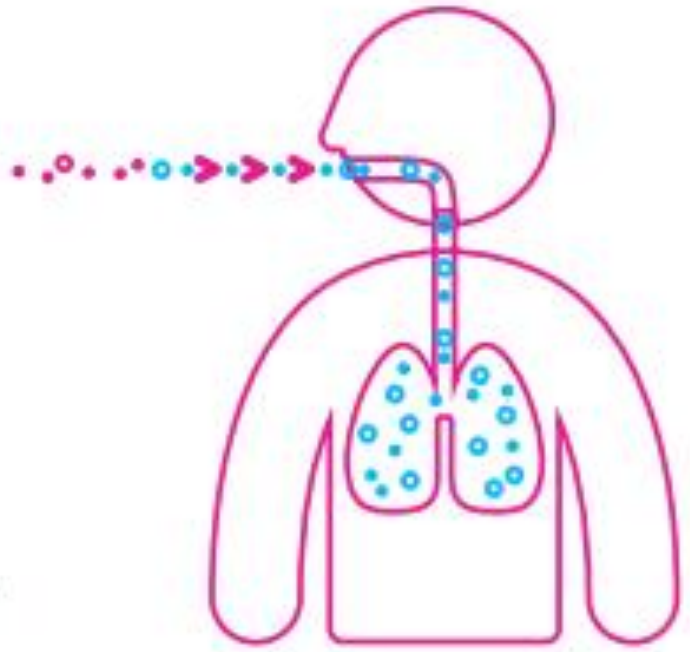

But there are things we can all do to help.

You can learn more about air pollution and find some simple ways to tackle it and protect your health at

cleanairhub.org.uk

Simple steps can have a big impact on the air your family breathes

Make travel choices for cleaner air

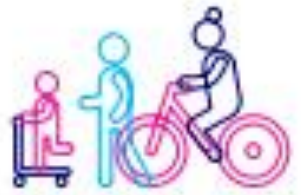

Use people power – Walk, scoot or cycle to school as often as you can.

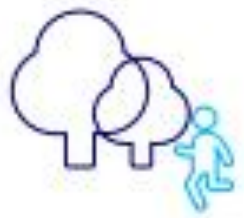

Discover the side streets – Use quieter streets when you're walking or on a bike to avoid the higher levels of air pollution on main roads.

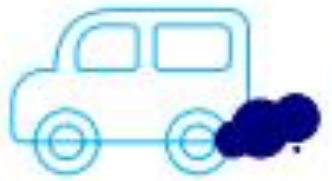

Don't idle – If you have to drive, turn off the engine when you are not moving, and it is safe to do so. Consider switching to an electric vehicle. Air quality can be worse inside the vehicle than outside.

Make cleaner air decisions in the home

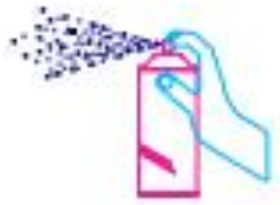

Use fragrance-free, milder cleaning products and avoid plug-in fragrances.

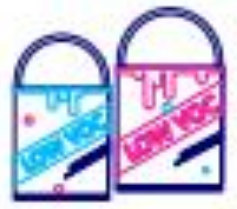

When decorating, choose safer paints and varnishes labelled 'low VOC' (volatile organic compounds).

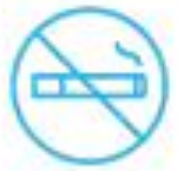

Ask people not to smoke in your home.

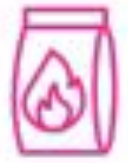

Reduce home burning as much as possible (e.g. log burners, coal fires or candles).

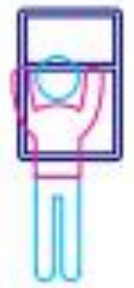

Ventilate your home - Open windows and use extractor fans when cooking or using cleaning products, but close windows near busy roads during rush hours.

Air Pollution and Children

Air pollution can worsen asthma symptoms including coughing, wheezing and breathlessness. The actions below can help:

1. Discover the side streets

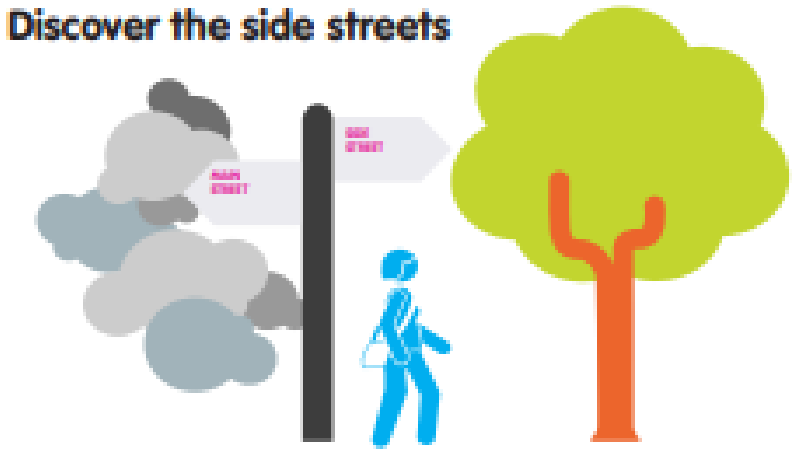

Use quieter roads and paths to keep away from heavy polluting traffic.

2. Leave the car behind

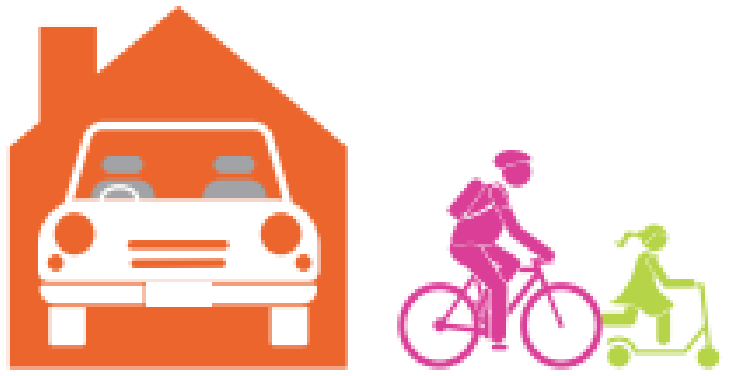

Encourage your whole family to walk, cycle and scoot more - air pollution can be higher inside a car than outside.

3. Turn the engine off

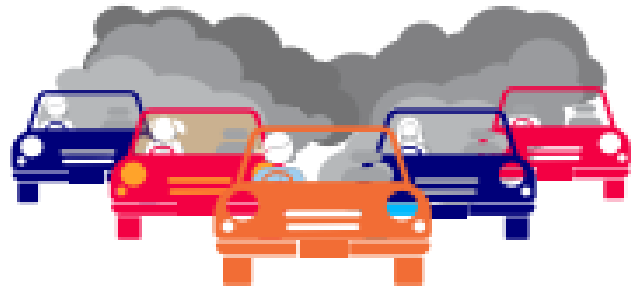

If you do need to use a car, ask the driver to turn the engine off when the car isn't moving.

4. Check the pollution forecast

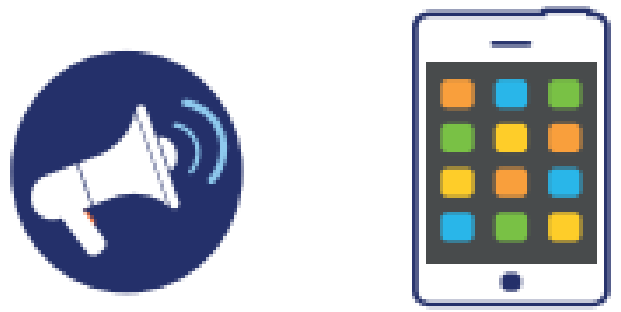

Check air pollution levels in your local area at uk-air.defra.gov.uk - this site provides hourly measurements and forecasts. Remember that air pollution increases significantly near busy roads, especially during rush hour.

5. Keep the air clean inside too

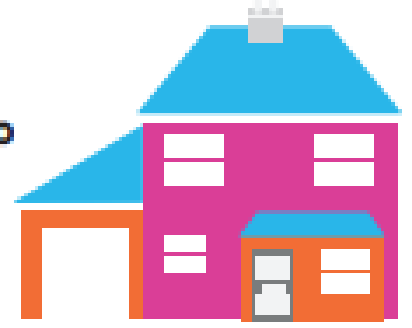

Use fragrance free and low-chemical cleaning products. Stop air pollution collecting in your home by using extractor fans and opening windows away from busy roads.

You can learn more about air pollution and find some simple ways to tackle it and protect your health at cleanairhub.org.uk

My air pollution plan:

Choose the actions that you and your family are going to do, when you can, to protect your health from air pollution:

	**Please tick all that apply**
We will walk, cycle or scoot to school 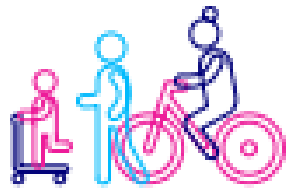	❑
We will use quieter routes to avoid roads with heavy traffic	❑
We will turn the engine off when the car is stationary and it is safe	❑
We will leave the car at home whenever we can 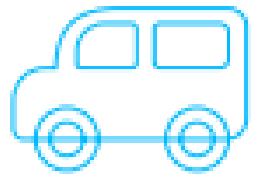	❑
I will use my inhaler as recommended by my GP or asthma nurse	❑
I will treat air pollution the same way I treat other asthma triggers	❑
We will buy fragrance-free, milder cleaning products and avoid plug-in fragrances	❑
When decorating, we will choose paints and varnishes labelled "low VOC"	❑
We will ask people not to smoke in our home 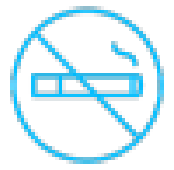	❑
We will avoid home burning as much as possible (e.g. log burners, coal fires or candles)	❑
We will turn on the extractor fan when cooking 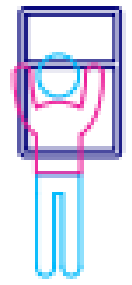	❑
We will open a window when cooking and cleaning	❑

global action plan

Department for Environment Food & Rural Affairs

Air Pollution and you

Air pollution affects us all from our first breath to our last, causing many health effects throughout our lives. Air pollution can worsen many long-term health conditions and cause respiratory and heart disease. It is linked with high blood pressure, strokes and dementia. It can also impact pregnancy and make people more likely to suffer infections like bacterial pneumonia and possibly COVID-19. The actions below can help:

1. Discover the side streets

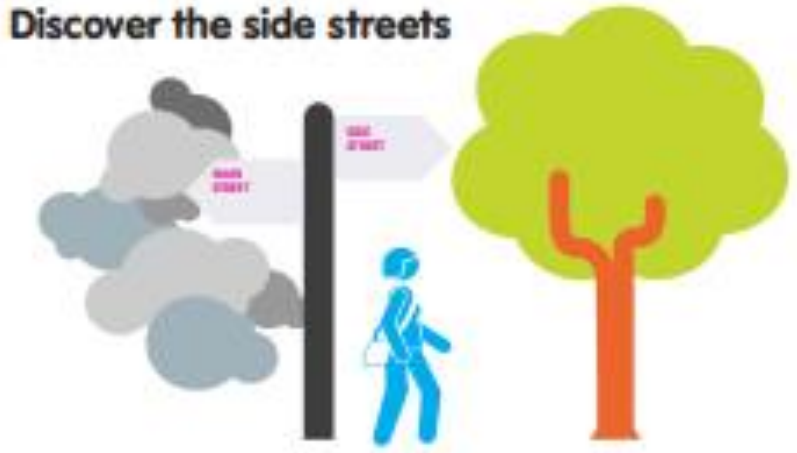

Use quieter roads and paths to keep away from heavy polluting traffic.

2. Leave the car behind

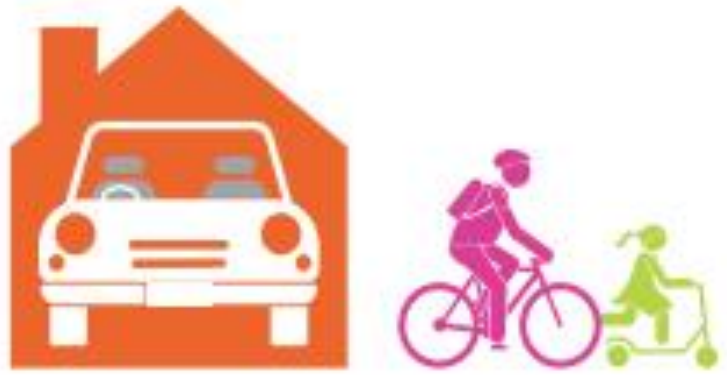

Encourage your whole family to walk, cycle and scoot more - air pollution can be higher inside a car than outside.

3. Turn the engine off

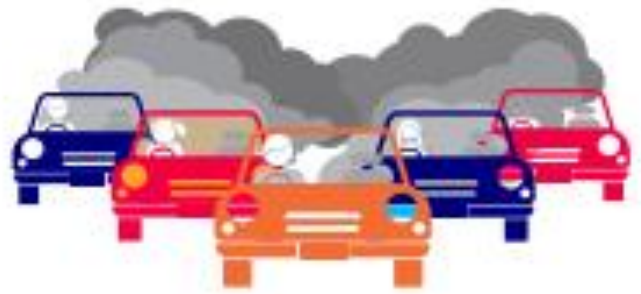

If you do need to use a car, ask the driver to turn the engine off when the car isn't moving.

4. Check the pollution forecast

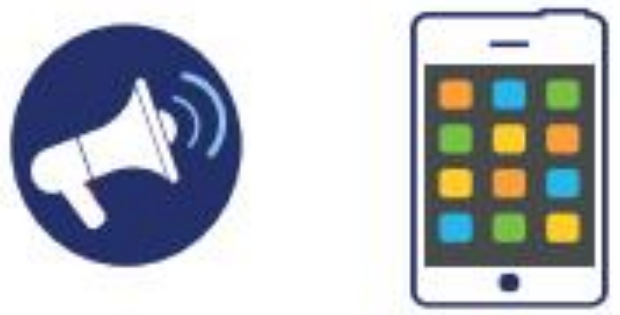

Check air pollution levels in your local area at uk-air.defra.gov.uk - this site provides hourly measurements and forecasts. Remember that air pollution increases significantly near busy roads, especially during rush hour.

5. Keep the air clean inside too

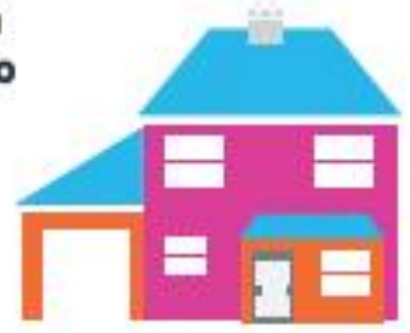

Use fragrance free and low-chemical cleaning products. Stop air pollution collecting in your home by using extractor fans and opening windows away from busy roads.

Air Pollution Checklist

Please check the actions you are going to do, when you can, to protect your health from air pollution:	**Please tick all that apply**
Leave the car at home whenever I can	❑
Walk and cycle more whenever I can	❑
Use quieter routes to avoid roads with heavy traffic	❑
Turn the engine off when the car is stationary and it is safe to do so	❑
Use inhalers as prescribed by my GP or practice nurse	❑
Buy fragrance-free, milder cleaning products and avoid plug-in fragrances	❑
Turn on the extractor fan when cooking	❑
Open the window when cooking and cleaning	❑
Avoid home burning as much as possible (e.g log burners, coal fires or candles)	❑
When decorating, choose paint labelled "low VOC"	❑
Ask people not to smoke in my home	❑

For more information on how air pollution can affect you, and how you can protect your health visit

www.cleanairhub.org.uk

global action plan

Department for Environment Food & Rural Affairs

Topline Messages for Patients

- Air Pollution seriously impacts your health
- There are things we can do to reduce it
- There are things we can do to reduce exposure

How to be a Clean Air Champion: 1

Leaflets and materials available

Speak with your first patients

What words and phrases work for you?

Be creative!

Make it personal

Develop your routine

How to be a Clean Air Champion: 2

- Introduce into your consultations
- Share with practice colleagues
- Speak at local education meeting(s)
- Speak at a regional meeting!
- GAP 3-month survey and interviews

Review of Learning Objectives

- Gain increased understanding of the health impacts of air pollution and what patients can do to protect their health.
- Understand how patients can avoid exposure and reduce contribution to air pollution, advising on potential behaviour changes.
- Consider patient engagement at different stages of patient pathway & see leaflets
- Knowing how to become a Clean Air Champion.

Winding up

- Reflections: The first step I will take on talking to patients about Clean Air is …?

Questions & Discussion

Thank you

@globalactplan

globalactionplan

globalactionplan

global-action-plan

Newsletter

global action plan

OUR LIVES. OUR PLANET.

Global Action Plan, Charity registered in England and Wales No. 1026148, in Scotland No. SC041260, registered company in England and Wales No. 2838296
