# Supplementary material for: Exploring the feasibility and acceptability of an educational intervention on air pollution for healthcare professionals
Source: Front Public Health. 2026 Aug 12;14:1862924. doi: 10.3389/fpubh.2026.1862924 (PMC13537046; doi:10.3389/fpubh.2026.1862924)
Supplement: Supplementary file 2 [file Data_Sheet_2.PDF]

Pre-Training Session Form

Q1. On a scale of 1-10, how would you rate **your understanding** of the health impacts of air pollution?

- ○ 0
- ○ 1
- ○ 2
- ○ 3
- ○ 4
- ○ 5
- ○ 6
- ○ 7
- ○ 8
- ○ 9
- ○ 10

Q2 On a scale of 1-10, how would you rate **your understanding** of what patients can do to protect their health?

- ○ 0
- ○ 1
- ○ 2
- ○ 3
- ○ 4
- ○ 5
- ○ 6
- ○ 7
- ○ 8
- ○ 9
- ○ 10

Q3 On a scale of 1-10, how would you rate **your confidence levels** in talking to your patients about air pollution?

- ◯ 0
- ◯ 1
- ◯ 2
- ◯ 3
- ◯ 4
- ◯ 5
- ◯ 6
- ◯ 7
- ◯ 8
- ◯ 9
- ◯ 10

Q4 On a scale of 1-10, how would you rate **your confidence levels** in giving your patients advice as to what they can do to reduce their exposure to air pollution.

- ○ 0
- ○ 1
- ○ 2
- ○ 3
- ○ 4
- ○ 5
- ○ 6
- ○ 7
- ○ 8
- ○ 9
- ○ 10

End of Block: default

Post-Training Session

Q1 On a scale of 1-10, how would you rate **your understanding** of the health impacts of air pollution?

- ○ 0
- ○ 1
- ○ 2
- ○ 3
- ○ 4
- ○ 5
- ○ 6
- ○ 7
- ○ 8
- ○ 9
- ○ 10

Q2 On a scale of 1-10, how would you rate **your understanding** of what patients can do to protect their health?

- ○ 0
- ○ 1
- ○ 2
- ○ 3
- ○ 4
- ○ 5
- ○ 6
- ○ 7
- ○ 8
- ○ 9
- ○ 10

Q3 On a scale of 1-10, how would you rate **your confidence levels** in talking to your patients about air pollution?

- ◯ 0
- ◯ 1
- ◯ 2
- ◯ 3
- ◯ 4
- ◯ 5
- ◯ 6
- ◯ 7
- ◯ 8
- ◯ 9
- ◯ 10

Q4 On a scale of 1-10, how would you rate **your confidence levels** in giving your patients advice as to what they can do to reduce their exposure to air pollution.

- ○ 0
- ○ 1
- ○ 2
- ○ 3
- ○ 4
- ○ 5
- ○ 6
- ○ 7
- ○ 8
- ○ 9
- ○ 10

Q5 On a scale of 1-10, how would you rate **your confidence levels** in acting as a 'Clean Air Champion?' (Spreading the learning to colleagues in your practice and beyond with the help of additional materials e.g., slides.)

- ◯ 0
- ◯ 1
- ◯ 2
- ◯ 3
- ◯ 4
- ◯ 5
- ◯ 6
- ◯ 7
- ◯ 8
- ◯ 9
- ◯ 10

Q6 Which parts of this session did you find most beneficial? Please order the following session's parts from most beneficial to least beneficial.

_______ The importance of talking about Air pollution including Jonathan Grigg introduction (4)
_______ S. Holgate Video - pathophysiology & epidemiology (5)
_______ Considering the patient pathway (6)
_______ GP cycling video (7)
_______ Messages to pass on to patients (8)
_______ How to be a clean air champion (9)

Q7 Is there anything we could do differently in future workshops/ sessions that might improve both your experience and the impact of your involvement?

Q8 Please agree or disagree with the following:

	Strongly Agree	Somewhat agree	Neither agree nor disagree	Somewhat disagree	Strongly disagree
There were enough teaching sessions for me to choose a day and time that suited my schedule (1)	○	○	○	○	○
The length of the teaching session was appropriate (1 hour) (2)	○	○	○	○	○
The topics covered were explained clearly (3)	○	○	○	○	○
The session objectives were met (4)	○	○	○	○	○

3-months Post Survey

Q1 Now that you have had the opportunity to use your learning from the training session, is there any other learning or information you would have liked to have been included?

____

Q2 In hindsight, how useful was the training in preparing you to speak to your colleagues about air pollution?

- ◯ Extremely useful
- ◯ Very useful
- ◯ Moderately useful
- ◯ Slightly useful
- ◯ Not at all useful

Q3 In hindsight, how useful was the training in preparing you to speak to your patients about air pollution?

- ◯ Extremely useful
- ◯ Very useful
- ◯ Moderately useful
- ◯ Slightly useful
- ◯ Not at all useful

Q4 Since your training session, how often have your used or referred back to the powerpoint slides from the training session?

- ○ Very frequently
- ○ Frequently
- ○ Occasionally
- ○ Rarely
- ○ Very rarely
- ○ Never

Implementing the pilot at work/with colleagues

Q5 What is the size of your team at your practice? (clinical and non-clinical)

____

Q6 Did you share the “clean air learning” with other members of your team?

- ○ Yes
- ○ No

Skip To: Q11 If Did you share the “clean air learning” with other members of your team? = No

Q7 How many members of your team were you able to speak to about the project?

____

Q8 Who did you talk to? (Tick all that apply)

- ○ GPs
- ○ Nurses
- ○ Administration staff
- ○ Pharmacists
- ○ Others, please tell us ______________________________

Q9 Did you find other ways to spread the clean air message (e.g., community meetings, education meetings outside of the practice)

- ○ No
- ○ Yes, please tell us ______________________________

GPs' Perspectives on Using/Sharing Materials

Q10 How helpful were the communications materials in enabling you to integrate/share information about air pollution with your team?

- ○ Extremely useful
- ○ Very useful
- ○ Moderately useful
- ○ Slightly useful
- ○ Not at all useful

Q11 How helpful were the communications materials in enabling you to integrate/share messages/information about air pollution with your patients?

- ◯ Extremely useful
- ◯ Very useful
- ◯ Moderately useful
- ◯ Slightly useful
- ◯ Not at all useful

Q12 From the adults' information leaflets which one did you find more useful as an information resource for you?

- ◯ Adult Long Leaflet
- ◯ Adult Health Checklist
- ◯ I didn't use the leaflets

Q13 From the adults' leaflets which one did you find more "patient" user friendly?

- ◯ Adult Long Leaflet
- ◯ Adult Health Checklist
- ◯ I didn't use the leaflets

Q14 From the children leaflets which one did you find more useful as an information resource for you?

- ◯ Children Long Leaflet
- ◯ Children Health Checklist
- ◯ I didn't use the leaflets

Q15 From the children leaflets which one did you find more “patient” user friendly?

- ◯ Children Long Leaflet
- ◯ Children Health Checklist
- ◯ I didn't use the leaflets

Q16 What resources did you find more useful?

- ◯ The paper version of the leaflets
- ◯ The electronic version of the leaflets
- ◯ I didn't use any of the leaflets

Q17 Did you use the poster/electronic resources provided for sharing on the practice information screens?

- ◯ Yes
- ◯ No

Q18 Did you encounter any barriers which prevented you from sharing information about air pollution with your patients?

- ◯ No
- ◯ Yes, please tell us ____

Q19 To what extent has your participation in the project helped you talk to your patients about **outdoor** air pollution with more confidence?

- ◯ To a great extent
- ◯ Somewhat
- ◯ Very little
- ◯ Not at all

Q20 To what extent has your participation in the project helped you talk to your patients about **indoor** air pollution with more confidence?

- ◯ To a great extent
- ◯ Somewhat
- ◯ Very little
- ◯ Not at all

Q21 Over the last two months, how often did you speak to your patients about air pollution?

- ◯ Very frequently
- ◯ Frequently
- ◯ Occasionally
- ◯ Rarely
- ◯ Very rarely
- ◯ Never

Q22 How receptive/interested did patients seem when it came to learning about air pollution and ways in which they could reduce exposure to harmful pollutants?

- ◯ Extremely receptive/interested
- ◯ Very receptive/interested
- ◯ Moderately receptive/interested
- ◯ Slightly receptive/interested
- ◯ Not at all receptive/interested

Q23 Thinking about the feedback that you received from your patients. What information did your patients found most valuable?

____

Q24 Do you have any particular example(s) that spring to mind where a patient was particularly receptive /interested?

____

Interview Topic Guide

Training session

1. Now that you have had the opportunity to use your learning from the training session, is there any other learning or information you would have liked to have been included?
2. In hindsight, how useful was the training in preparing you to speak to your colleagues about air pollution?
3. In hindsight, how useful was the training in preparing you to speak to your patients about air pollution?
4. Since your teaching session, how often have your used or referred back to the powerpoint slides from the teaching session? If yes, why?

Implementing the pilot at work/with colleagues / assessment of whether the 'Champion' model worked

1. To what extent were you able to engage your team in the project? I.e. How many colleagues were you able to inform about the project, and to what degree do you think they were able to adopt this information into their practice?
2. Did your colleagues recognise air pollution as an issue or not? If they were not open to hear about it, were reasons given?
3. If this project was rolled out nationally, what do you think would be the most useful ways to engage colleagues on the topic?
4. Where do you think are the most important pathways in terms of training for healthcare professionals where learning on air pollution could be included?
5. Do you have any examples of when you were able to share information on air pollution outside of your practice, or in situations outside of your normal work environment?

Health Professional's Perspectives on Using/Sharing Materials

1. How helpful were the communication materials in enabling you to integrate/share information about air pollution with your team? Did you find the presentation slides provided of particular use?
2. How helpful were the communications materials in enabling you to integrate/share messages and information about air pollution with your patients? Did you find leaflets (electronic or physical), files for social media and electronic screens, or suggested text for emails and texts to patients of particular use?
3. Where there any logistical or physical challenges in terms of handing or sharing the leaflets?
4. If this project was rolled out nationally, are there any changes you would suggest to the types of materials we use and where they are shared?

Health Professionals Perspectives on Speaking about Air Pollution to patients

1. What were your experiences of sharing information on air pollution during the patient pathway?
2. What were the biggest opportunities in terms of having conversations about air pollution?
3. What would you say are the biggest barriers to speaking to your patients on air pollution? What factors hindered the delivery of information to patients?
4. Thinking about the feedback that you received from your patients. What information did your patients found most valuable?
5. How did your patients respond to the messages in the communications materials? Did your patients provide any feedback on the leaflet that you gave them?
6. Have you seen or heard of the information you gave to patients having an impact their behaviour or their intended behaviours?
7. Do you have any particular example(s) that spring to mind where a patient was particularly receptive /interested?

………………………………………………….

Last Question! Do you have any other reflections, ideas or instances that you would like us to know about?
