# Supplementary material for: Exploring the feasibility and acceptability of an educational intervention on air pollution for healthcare professionals
Source: Front Public Health. 2026 Aug 12;14:1862924. doi: 10.3389/fpubh.2026.1862924 (PMC13537046; doi:10.3389/fpubh.2026.1862924)
Supplement: Supplementary file 3 [file Data_Sheet_3.PDF]

Supplementary File 3. Additional illustrative quotations by theme

**Perceive usefulness**	*"It was very useful in laying out the broad framework, but obviously following on from that it just takes a certain amount of time to develop the skills and get used to building it into your own clinical practice. I'm still in the process of doing that" GP_1*
**Communication Challenges**	*"I found it was much easier to talk about air pollution when it appeared at face value to be moved directly related to what the patient had come in with. So, if I was speaking to a patient whose COPD wasn't well controlled, I could talk about air pollution, similarly with asthma. Or maybe somebody with pregnancy or a baby check, it seemed very relevant to a small baby with developing lungs. But if somebody, I don't know, coming with knee pain or something random, it would be much harder to bring it into the conversation…" GP_9*<br><br>*"[…] a person doing it for their child, I think they were a lot more receptive […] I am nurturing and caring for this individual. I'm doing what I can to keep them out of harm. You've given me some practical things that I can go and do […] you know, reduce the amount of harm that they're suffering" GP_3*
**Patient receptivity**	*"Some of the conversation was not just about how to avoid air pollution, but how to reduce their contribution to air pollution. They didn't want to be the ones who were contributing to other people's suffering as well" GP_3*<br><br>*"Couple of patients who have asthma and had more frequent exacerbations recently […] were quite receptive to thinking about how even just things like you know, trying to walk on different streets at different times and trying to just reduce their direct risk to air pollution […] those kinds of practical things and understanding a bit more about how their asthma classifications and air pollution are potentially linked" GP_6*<br><br>*"It's important to be able to give them. You know, practical realistic advice about how they can reduce their exposure" GP_1*

**Practical educational needs**	*"If you're busy and there's lots of things going on, then there can just be a lack of time and that's why having written information you can send is really useful, because then you can quickly bring it up […] and signpost people to more information"* GP_6
**Structural and system barriers**	*"I think that if we're having any training on asthma and COPD, air quality and air pollution should be included in that. So that then it just becomes a kind of standard education"* GP_6<br><br>*"I think the problem is that we're all given so much to read all the time […]. It needs to be looked at from a positive angle, rather than an awful doom and gloom, and be included in business as usual"* GP_2
